# A robust and validated method for the determination of 21 urinary metabolites of 15 plasticizers, including phthalates, DEHTP, and DINCH, by online SPE and liquid chromatography-tandem mass spectrometry

**DOI:** 10.1007/s00216-026-06634-8

**Published:** 2026-07-04

**Authors:** Rosa Mercadante, Simone Cafagna, Marco Rizzo, Elisa Roggia, Silvia Fustinoni

**Affiliations:** 1 Dipartimento di Scienze Cliniche e di Comunità, Dipartimento di Eccellenza 2023-2027, Università degli Studi di Milano, Milano, Italy; 2 Toxicology Lab Fondazione IRCCS Ca’ Granda Ospedale Maggiore Policlinico, Milan, Italy

**Keywords:** Metabolites of phthalates, Terephthalate, DINCH, Urine, LC-MS/MS, Biological monitoring

## Abstract

Phthalates and their alternative plasticizers terephthalates and hexahydrophthalates are widely used in consumer products, leading to continuous human exposure and potential risk for health. The aim of this study was to develop an analytical assay for the quantification of urinary metabolites of these plasticizers as biomarkers of exposure. Twenty-one urinary metabolites from 15 plasticizers, including 13 phthalates, one terephthalate (di-2-ethylhexyl terephthalate, DEHTP), and one hexahydrophthalate (di-isononyl cyclohexane-1,2-dicarboxylate, DINCH), were deconjugated by enzymatic hydrolysis, separated, and quantified using liquid chromatography-triple quadrupole mass spectrometry (LC-MS/MS) in the presence of 17 isotopically labeled internal standards. A full validation, according to international guidelines, was performed and an external proficiency test was undergone. Forty-six urine samples collected from adults belonging to the Italian general population were assayed to test the method. The analytes, after enzymatic deconjugation, were enriched by online solid phase extraction (SPE); the interferences from contaminants in the mobile phase were delayed with pre-analytical columns and the chromatography was successfully separating all analytes. The assay was precise (% relative standard deviation within 15.6%), accurate (% relative to theoretical value between 96.5 and 108.5%) and sensitive, with lower limits of quantification (LLOQ) in the range 0.10 to 0.78 µg/L. Biases arising from matrix effects and long-term storage had a negligible impact on accuracy, with recoveries remaining within 84–111%. The external proficiency exercise was successful across multiple rounds. In the study sample, the most abundant analytes were monoethyl phthalate (MEP), mono(2-ethyl-5-carboxypentyl) terephthalate (5-cx-MEPTP), monobutyl phthalate (MnBP) and monoisobutyl phthalate (MiBP) (38, 11, 8, and 8 µg/L), metabolites of diethyl phthalate (DEP), DEHTP, di-*n*-butyl phthalate (DnBP), and di-isobutyl phthalate (DiBP), respectively. The developed method demonstrates good performances, with accurate results and high throughput; it is well-suited for large-scale biomonitoring studies in the general population.

**Graphical abstract:**

**Figure:**
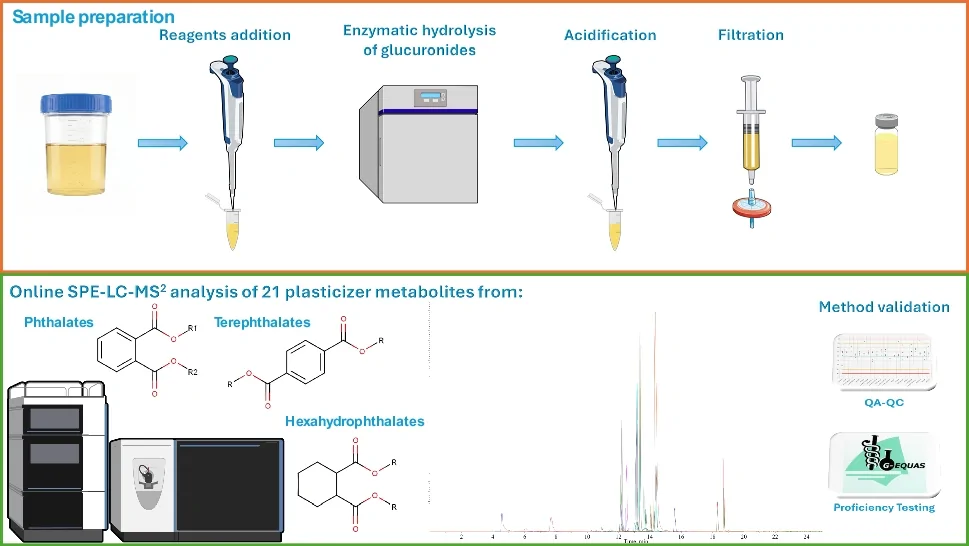

**Supplementary Information:**

The online version contains supplementary material available at https://doi.org/10.1007/s00216-026-06634-8.

## Introduction

The industrial use of plasticizers is intended to modify the physico-mechanical properties of polymeric materials, thereby enhancing their ductility and flexibility. The plasticization mechanism relies on the intercalation of additive molecules between polymer chains, with the final effect of a more elastic and deformable material [1].

The most widely used plasticizers include alkyl- and aryl-diester derivatives of phthalic anhydride (phthalates), terephthalic acid (di-2-ethylhexyl terephthalate, DEHTP), and hexahydrophthalic anhydride (di-isononyl cyclohexane-1,2-dicarboxylate, DINCH).

High molecular weight phthalates (side chains containing at least 7 carbon atoms) are primarily used in the production of flexible polyvinyl chloride (PVC). By contrast, low molecular weight phthalates have diverse applications that extend beyond plasticization; among these, diethyl phthalate (DEP) is used in personal care products. DEHTP and DINCH have been developed as alternative plasticizers to replace specific high molecular weight phthalates whose use is now restricted [2, 3].

Since these plasticizers are not chemically bound to the polymer matrix, they can migrate leading to contamination of food and of the surrounding media. Human exposure occurs via oral, inhalation, and dermal routes, the latter being particularly relevant for low molecular weight phthalates [3–5].

Once absorbed, phthalates, terephthalates, and hexahydrophthalates are rapidly metabolized to monoesters and, in the case of higher molecular weight compounds, further oxidized; the resulting metabolites, mainly in the glucuronidated form, are excreted in urine [6].

Some phthalates are recognized as toxics with endocrine-disrupting properties, representing a major public health concern because of their effects on reproductive health, neurodevelopment, and metabolic disorders [4, 7, 8]. Phthalates such as di-*n*-butyl phthalate (DnBP) and butyl benzyl phthalate (BBzP) have been found to be linked to a deterioration in semen quality [9, 10]. In addition, exposure to di-2-ethylhexyl phthalate (DEHP), di-isononyl phthalate (DiNP), and di-isodecyl phthalate (DiDP) has been associated with impaired female fertility [11, 12].

Due to their reproductive toxicity and ability to interfere with the endocrine system, many phthalates are regulated by the European Union regulation Registration, Evaluation, Authorization and Restriction of Chemicals (REACh) as substances of very high concern (DEHP, di-isobutyl-phthalate, DiBP, DnBP, BBzP, di-cyclohexylphthalate, DCHP, di-*n*-pentyl phthalate, DnPP and di-*n*-hexyl phthalate, DnHxP); some are restricted (DEHP, DiBP, DnBP, di-isononyl phthalate, DiNP, BBzP, DiDP, and di-n-octylphthalate, DnOP are included in annex XVII) or under evaluation, particularly regarding endocrine-disrupting (diethyl phthalate, DEP, and di-2-propylheptyl phthalate, DPHP) and persistent, bioaccumulable and toxic (PBT) properties (DnBP) [13, 14]. Additionally, DiBP is recognized as PBT, while DnBP, BBzP, and DnPP are classified as harmful to the environment [15]. According to current epidemiological evidence, the plasticizers DINCH and DEHTP are regarded as safer alternatives to traditional phthalates and are not classified as hazardous according to the CLP regulation [16, 17]. European biomonitoring data show that exposure to regulated and non-regulated phthalates is decreasing, while an increase in exposure to DEHTP and DINCH is being observed [18]; a similar trend has also been reported in the US [19].

Although plasticizers are metabolized and eliminated rapidly [6, 20, 21], human exposure to these compounds should be regarded as continuous; the quantification of urinary metabolites represents the preferred approach for assessing this exposure [22].

For the analysis of plasticizer metabolites in urine, several analytical assays, based on liquid chromatography-tandem mass spectrometry (LC-MS/MS), have previously been published [23–29]. However, the analytical determination of these metabolites remains challenging and new updated methods are desirable. The main reasons are (1) The spectrum of phthalates and alternative plasticizers used in consumer products is continuously evolving. Regulatory restrictions on specific compounds have led to their progressive replacement with structurally related alternatives, resulting in the introduction of new chemicals whose metabolites may not be included in previously validated analytical panels. Consequently, analytical methods must be periodically updated to reflect changes in market composition and human exposure patterns. (2) For certain plasticizers, as in the case of DiNP and DiDP, the presence of positional isomers gives rise to clusters of partially overlapping chromatographic peaks [25, 30, 31]. These isomeric metabolites often exhibit similar fragmentation patterns, complicating their mass-chromatographic resolution and quantitative determination. Therefore, objective and standardized integration criteria are required to ensure consistent quantification. (3) Ubiquitous contamination by phthalates and their metabolites represents a significant analytical concern. These compounds may be present in laboratory equipment, solvents, and water systems [32], contributing to elevated background signals and potentially compromising quantitative reliability, particularly at low concentration levels. In the present method, the use of two pre-analytical columns was introduced to eliminate these contaminants from chromatograms. (4) Our method has a high throughput, with limited sample handling and high automation, thanks to the use of online SPE. (5) The method has been submitted to extensive validation, and the results warrant good analytical performance.

Here, we present an LC-MS/MS method for the quantification of 21 urinary metabolites of 15 plasticizers, including phthalates, DEHTP, and DINCH (Table 1), to take into consideration the current use of plasticizers. The method, taking advantage of the published experiences, aims to overcome the previously mentioned issues and to offer an improved assay for biomonitoring exposure of the general population. The method benefits from a set of 17 internal standards, ensuring robust correction across a wide range of analytes, and from an optimized sample preparation based on online SPE, which simplifies and accelerates sample processing compared to manual cartridge-based methods, reducing potential for errors and improving reproducibility. The method was rigorously validated in accordance with the international guidelines, verified for accuracy through an external quality assurance exercise, and ultimately applied to a small sample from the adult Italian general population.

**Table 1:** Phthalates and phthalate substitutes and their urinary metabolites measured in the present method, with CAS numbers and abbreviations

Phthalates/phthalate substitutes (parent compounds)	Parent compound abbreviation	Parent compound CAS number	Urinary metabolite (analytes)	Analyte abbreviation	Analyte CAS number
Dimethyl phthalate	DMP	131-11-3	Monomethyl phthalate	MMP	4376-18-5
Diethyl phthalate	DEP	84-66-2	Monoethyl phthalate	MEP	2306-33-4
Di-n-butyl phthalate	DnBP	84-74-2	Monobutyl phthalate	MnBP	131-70-4
Di-isobutyl phthalate	DiBP	84-69-5	Monoisobutyl phthalate	MiBP	30833-53-5
Butyl benzyl phthalate	BBzP	85-68-7	Monobutyl phthalate	MnBP	131-70-4
			Monobenzyl phthalate	MBzP	2528-16-7
Di-n-pentyl phthalate	DnPP	131-18-0	Mono-n-pentyl phthalate	MnPP	24539-56-8
Di-n-hexylphthalate	DnHxP	84-75-3	Mono-n-hexyl phthalate	MnHxP	24539-57-9
Di-cyclohexyl phthalate	DCHP	84-61-7	Monocyclohexyl phthalate	MCHP	7617-36-4
Di-n-octyl phthalate	DnOP	117-84-0	Mono-n-octyl phthalate	MnOP	5393-19-1
Di-2-ethylhexyl phthalate	DEHP	117-81-7	Mono(2-ethylhexyl) phthalate	MEHP	4376-20-9
			Mono(2-ethyl-5-hydroxyhexyl) phthalate	5-OH-MEHP	40321-99-1
			Mono(2-ethyl-5-carboxypentyl) phthalate	5-cx-MEPP	40809-41-4
			Mono(2-ethyl-5-oxohexyl) phthalate	5-oxo-MEHP	40321-98-0
Di-isononylphthalate	DiNP	28553-12-0	Mono(4-methyl-7-hydroxyoctyl) phthalate	OH-MiNP	936021-98-6
			Mono(4-methyl-7-carboxyheptyl) phthalate	cx-MiNP	936022-02-5
Di-isodecyl phthalate	DiDP	26761-40-0	Mono(2,7-dimethyl-7-carboxyheptyl) phthalate	cx-MiDP	1373125-93-9
Di-2-propylheptyl phthalate	DPHP	53306-54-0	Mono(2-propyl-6-hydroxyheptyl) phthalate	OH-MPHP	1372605-11-2
Di-2-ethylhexyl terephthalate	DEHTP	6422-86-2	Mono(2-ethyl-5-hydroxyhexyl) terephthalate	5-OH-MEHTP	1684398-38-6
			Mono(2-ethyl-5-carboxypentyl) terephthalate	5-cx-MEPTP	1684398-42-2
Di-isononyl cyclohexane-1,2-dicarboxylate	DINCH	166412-78-8	2-(((Hydroxy-4-methyloctyl)oxy)carbonyl)cyclohexanecarboxylic acid	7-OH-MINCH	1637562-52-7
			2-(((Hydroxy-4-methyloctyl)oxy)carbonyl)cyclohexanecarboxylic acid	7-oxo-MINCH	1588520-62-0

## Materials and methods

### Chemicals

Table 2 lists the 21 analytes and their corresponding isotopically labeled internal standards, along with their abbreviations, purity, molecular structures (exported from MarvinSketch, ver. 24.2.2, ChemAxon) [33], and Chemical Abstract Service (CAS) registry numbers. The optimized instrumental conditions for the mass spectrometric detection of multiple reaction monitoring (MRM) transitions and product ion structures are also reported.

**Table 2:**
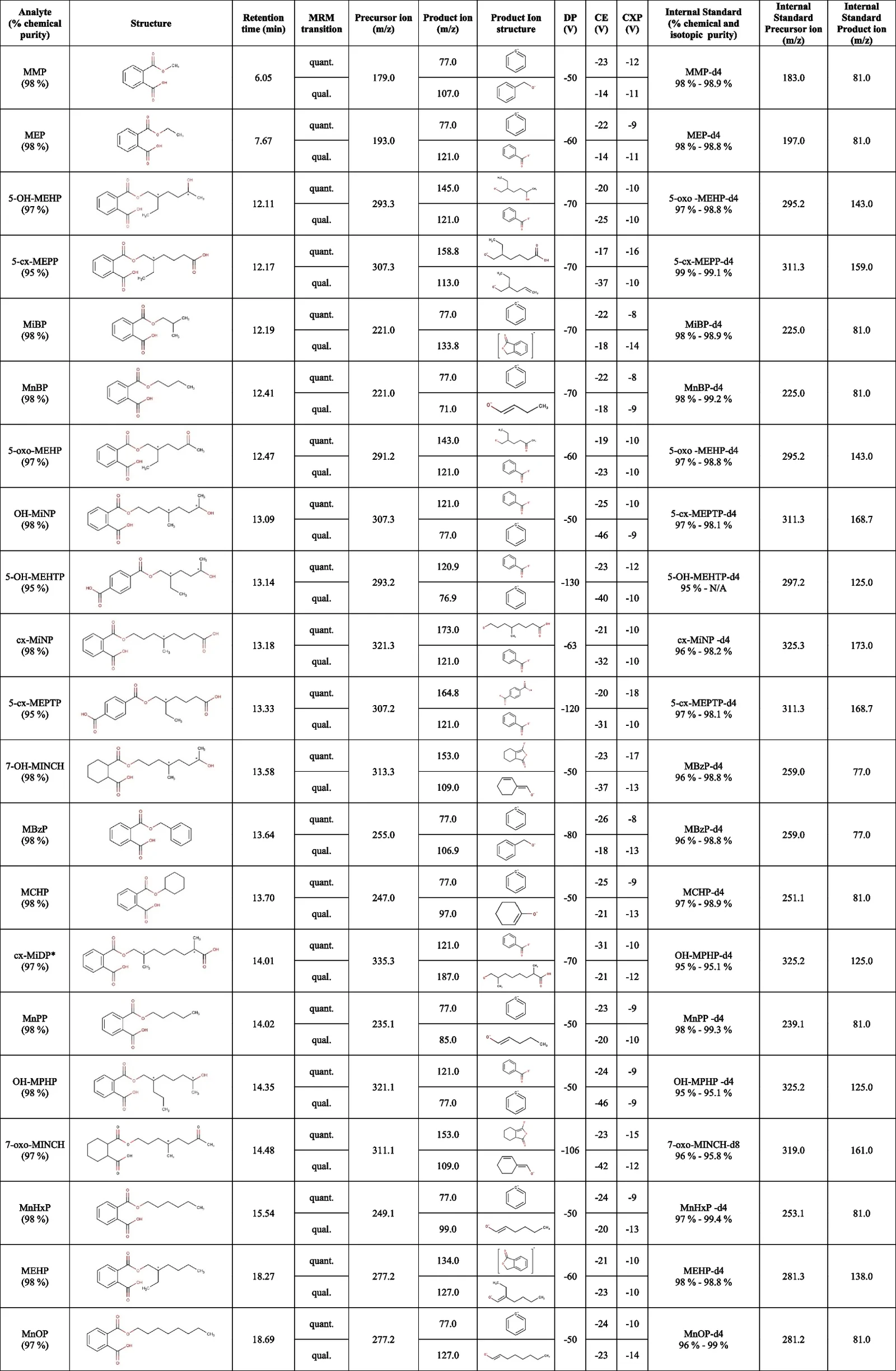
List of the analytes with their chemical structures, retention times, and MS/MS instrumental settings. For each analyte, the MRM transitions used for the quantitation (quant.) and the qualification (qual.) are given as precursor and product ions; proposed chemical structures for each product ion are also reported. The declustering potential (DP), the collision energy (CE), and the collision exit potential (CXP) applied to each transition are provided; the entrance potential (EP) is fixed at −10 V for all transitions. For each analyte, the corresponding isotopically labeled internal standard is reported, along with its purity, and specific precursor/product ion transitions

*An additional qualifier transition 321/77 was monitored

*N/A* not available on certificate of analysis

Product ion structures were drawed with MarvinSketch, ver. 24.2.2, ChemAxon

The chemicals 5-OH-MEHTP, 5-cx-MEPTP, 5-cx-MEPP-d4, 5-cx-MEPTP-d4, and 5-OH-MEHTP-d4 were obtained from Cansyn Chem Corp. (Toronto, Canada), while MnHxP was supplied by abcr Gute Chemie (Karlsruhe, Germany). All the other native compounds, the internal standards, and the probe used for testing hydrolysis efficiency 4-methylumbelliferyl-β-D-glucuronide (4-MUG, CAS: 6160-80-1) and the deconjugated, isotopically labeled 4-methylumbelliferone-^13^C_4_ (4-MU-^13^C_4_, CAS: 1569304-40-0) were purchased from Toronto Research Chemicals (North York, Canada).

For sample and solution preparation, analytical-grade acetonitrile, ammonium acetate, formic acid, glacial acetic acid, and methanol, along with the 140 U/mL β-glucuronidase solution (from *Escherichia coli* K12), were purchased from Sigma-Aldrich (Milan, Italy).

Ultrapure water was produced by a Rephille purification system (Roma, Italy).

### Standard, calibration, and quality control solutions

The neat standards were solubilized in methanol directly in the commercial vial to obtain the stock solutions at 1 mg/mL, which were stored at −20 °C. A multi-analyte working solution, named MIX PHTs (5000 µg/L in methanol), was prepared by combining the individual stock solutions. Similarly, an internal standard working solution (MIX IS PHTs) was prepared in ultrapure water at the concentrations detailed in Supplementary Table 1.

Matrix-matched calibration standards (ranging from 0.10 to 50 µg/L) and quality control (QC) samples at three levels (low, QCL; medium, QCM; and high, QCH; concentrations in Table 5) were prepared by spiking pooled urine obtained from healthy volunteers. This pool was prepared by mixing equal volumes of urine from several adult Italian donors (males and females in equal proportions), selected for their low concentrations of the target analytes.

**Table 3:** Percentage of glucuronide conjugates and matrix effect in urine samples from six individuals: the matrix effect results are given as relative standard deviation of the slopes (% RSD slope) of six calibration curves and back calculate concentrations, relative standard deviations (% RSD Calc. Conc.), and % accuracy of three different levels of the calibration curve

Analyte	% of glucuronide conjugates *n* = 6	% RSD slope *n* = 6	Level	Concentration (µg/L)	% RSD calc. conc *n* = 6	% accuracy ± SD *n* = 6
MMP	N/A	4.4	1	3.13	18.8	108 ± 20
			2	12.50	11.6	97 ± 11
			3	50.00	4.6	101 ± 5
MEP	28.2 ± 9.2	4.8	1	6.25	19.1	93 ± 18
			2	25.00	9.3	97 ± 9
			3	50.00	4.9	100 ± 5
5-OH-MEHP	96.9 ± 0.9	9.3	1	0.78	13.9	107 ± 15
			2	6.25	13.6	98 ± 13
			3	50.00	9.8	101 ± 10
5-cx-MEPP	48.3 ± 12.9	5.1	1	0.78	4.9	104 ± 5
			2	6.25	5.3	102 ± 5
			3	25.00	4.8	101 ± 5
MiBP	94.5 ± 2.9	3.5	1	1.56	3.8	99 ± 4
			2	6.25	6.7	98 ± 7
			3	50.00	2.6	101 ± 3
MnBP	95.9 ± 1.7	6.8	1	3.13	25.3	84 ± 21
			2	12.50	5.3	94 ± 5
			3	50.00	6.6	99 ± 7
5-oxo-MEHP	96.1 ± 1.6	8.0	1	0.20	5.1	92 ± 5
			2	1.56	4.3	99 ± 4
			3	25.00	7.1	101 ± 7
OH-MiNP	91.1 ± 7.3	5.1	1	0.39	15.1	90 ± 14
			2	3.13	10.7	100 ± 11
			3	25.00	5.4	103 ± 6
5-OH-MEHTP	59.3 ± 33.0	5.0	1	0.20	17.7	97 ± 17
			2	1.56	3.9	102 ± 4
			3	25.00	6.8	98 ± 7
cx-MiNP	39.0 ± 12.2	4.1	1	1.56	15.4	102 ± 16
			2	6.25	4.8	103 ± 5
			3	25.00	6.9	103 ± 7
5-cx-MEPTP	7.0 ± 5.7	6.1	1	3.13	17.9	111 ± 20
			2	12.50	6.2	103 ± 6
			3	50.00	7.3	99 ± 7
7-OH-MINCH	100.0	4.6	1	0.78	6.6	89 ± 6
			2	6.25	5.1	96 ± 5
			3	50.00	4.6	102 ± 5
MBzP	97.5 ± 0.8	13.9	1	0.39	22.2	102 ± 23
			2	3.13	13.1	95 ± 13
			3	50.00	15.4	101 ± 16
MCHP	N/A	7.8	1	0.39	11.5	98 ± 11
			2	3.13	12.1	97 ± 12
			3	50.00	8.1	102 ± 8
cx-MiDP	N/A	13.6	1	0.78	16.7	104 ± 17
			2	6.25	16.3	95 ± 15
			3	50.00	14.2	100 ± 14
MnPP	N/A	3.5	1	0.20	5.0	94 ± 5
			2	3.13	7.7	104 ± 8
			3	50.00	3.6	100 ± 4
OH-MPHP	N/A	2.5	1	0.20	6.5	104 ± 7
			2	3.13	2.0	102 ± 2
			3	50.00	7.2	97 ± 7
7-oxo-MINCH	98.1 ± 1.6	2.0	N/A	N/A	N/A	N/A
MnHxP	N/A	2.7	1	0.20	7.1	102 ± 7
			2	3.13	9.3	99 ± 9
			3	50.00	3.7	101 ± 4
MEHP	88.3 ± 8.3	1.8	1	0.78	14.7	96 ± 14
			2	6.25	2.0	99 ± 2
			3	50.00	2.4	99 ± 2
MnOP	N/A	1.9	1	0.20	10.2	86 ± 9
			2	3.13	5.2	92 ± 5
			3	50.00	3.0	100 ± 3

*N/A* data not available

Regarding the enzymatic hydrolysis procedure, a 1:5 dilution of β-glucuronidase in ammonium acetate water solution (pH 6.0–6.5) was freshly prepared before use (enzyme solution).

### Equipment

The LC-MS/MS instrumentation was an Agilent Technologies chromatograph featuring a 1260 analytical binary pump (Cernusco sul Naviglio, Italy) coupled with an ESI source-equipped Sciex QTRAP 5500 hybrid triple quadrupole/linear ion trap mass spectrometer (Monza, Italy). Online SPE was performed using a dedicated setup integrated with the chromatographic system. This configuration featured a quaternary pump (loading pump) and a pair of ten-port time-controlled valves.

A Poroshell 120 EC-C18 (50 × 4.6 mm, Agilent Technologies) was placed between the binary pump and the injector and a Hypersil GOLD PFP (50 × 2.1 mm, 3 µm, Thermo Scientific) was placed downstream of the loading pump to delay plasticizers metabolites (MMP, MEP, MiBP, MnBP, and MEHP) present as contaminants in the mobile phase [32].

Online SPE was optimized testing different cartridges, including Strata-X (oxopiperidine-functionalized polystyrene, 20 × 2.0 mm, 25 µm, Phenomenex) and the X-Bridge (C18, 30 × 2.1 mm, 10 µm, Waters); however, the PLRP-S (polystyrene-divinylbenzene, 12.5 × 2.1 mm, 15–20 µm, Agilent Technologies) was chosen as it provided the best performance. Chromatographic separation was achieved using an Accucore Phenyl-X (150 × 2.1 mm, 2.6 µm, Thermo Scientific) protected by a matching guard column Accucore Phenyl-X (10 × 2.1 mm, 2.6 µm, Thermo Scientific).

Sample filtration was performed manually using regenerated cellulose syringe filters (13 mm, 0.22 µm).

### Online SPE LC-MS/MS analysis

The autosampler temperature was kept at 10 °C. As detailed in Table 4, 100 µL of the sample was loaded onto the cartridge (kept at room temperature) at a flow rate of 1.0 mL/min using 80% of eluent A_q_ (0.125% formic acid in water) and 20% of eluent B_q_ (methanol). After 2.5 min of enrichment and clean-up step, the SPE valve was switched from the “loading” to the “elution” position. The analytes were then transferred to the analytical column (30 °C) and separated by the analytical pump at 0.4 mL/min using a gradient of eluent A_b_ (0.1% formic acid in water) and B_b_ (0.1% formic acid in acetonitrile) (Table 4). The total analysis time was 25 min.

**Table 4:** Online SPE and LC settings with times and percentage of the different mobile phases for the quaternary pump A_q_ and B_q_ (Loading) and the binary pump A_b_ and B_b_ (Elution), SPE valve positions, and flow rates

Quaternary pump (loading)				SPE valve		Binary pump (elution)			
Time (min)	% A_q_ (0.125% formic acid in H_2_O)	% B_q_ (methanol)	Flow rate (mL/min)	Time (min)	Position	Time (min)	% A_b_ (0.1% formic acid in H_2_O)	% B_b_ (0.1% formic acid in acetonitrile)	Flow rate (mL/min)
0.0–3.0	80	20	1.0	0.0–2.5	Loading	0.0–3.5	80	20	0.4
3.5–19.0	50	50	0.3	2.5–20.0	Elution	10.5–14.0	55	45	
19.5–22.0	0	100	1.0	20.0–25.0	Loading	17.0–20.0	0	100	
22.1–23.5	80	20	0.3			20.1–25.0	80	20	
24.0–25.0	80	20	1.0						

The mass spectrometer operated in the scheduled MRM under negative ionization, with a target scan time set at 0.4 s. MS/MS transitions were identified through direct infusion of diluted stock solutions (in methanol) and optimal collision energies were selected based on breakdown curves. This procedure also allowed the optimization of the collision exit potential and the nitrogen pressure of collision cell, that was set to “low.” Through the flow injection analysis, the optimal declustering potential for each compound was determined, and the source parameters were optimized and set as follows: gas 1 (air) pressure 60 psi, gas 2 (air) pressure 70 psi, curtain gas (N_2_) pressure 45 psi, heater temperature 650° C, ion spray voltage −4500 V. Quantifier (quant.) and qualifier (qual.) transitions were selected based on the analysis of spiked urine, prioritizing maximum signal intensity and minimal matrix interference. For each isotopically labeled internal standard, the MS/MS transition was set to correspond directly to the quantifier transition of its respective unlabeled analyte.

The target analytes were identified based on their retention times and specific MRM transitions, as detailed in Table 2, which provides the chemical structures for both the analytes and their characteristic product ions [34]. Analyst® (version 1.7.2; AB Sciex, Monza, Italy) and Analyst Device Driver softwares were used for instrument control and batch preparation. Peak integration and result verification were handled by MultiQuant™ software (version 3.0.3; Sciex, Monza, Italy), followed by data processing and calculation with Microsoft Excel.

### Sample preparation

For each unknown sample, a 400 µL aliquot was transferred into a 1.5-mL conical tube, to which 40 µL of MIX IS PHTs, 80 µL of ultrapure water, and 40 µL of enzyme solution were added. After adding 240 µL of aqueous ammonium acetate solution (1 M, pH = 6.0/6.5), the sample was vortexed and placed in an oven at 37° for 1 h for the hydrolysis step. To quench the enzymatic activity, 400 µL of 3.75 M acetic acid aqueous solution (21.5% v/v, quenching solution) was added, decreasing the pH to 4.0. The sample was vortexed and filtered directly into the vial for analysis. The same procedure was followed for the preparation of blank, calibration, and QC samples, omitting the hydrolysis step. In these cases, the enzyme solution was replaced with 40 µL of aqueous ammonium acetate solution (1 M, pH = 6.0/6.5). The ubiquitous presence of plasticizer metabolites in human urine complicates the use of hydrolyzed matrix pools for calibration and quality control purposes. Such backgrounds would hinder the accurate discrimination of spiked analytes from the native signals; consequently, the pooled urine used for calibrators and QCs was not subjected to enzymatic hydrolysis. An example of background concentrations in pooled urine not subjected to enzymatic hydrolysis is reported in Supplementary Table 2.

### Analytical sequence

In routine analysis, the calibration curve and the QCs were included with every batch of unknown samples. In addition, approximately 10% of the samples were prepared and analyzed in duplicate, to monitor the repeatability. Each analytical sequence began with the acquisition of the calibration standards, followed by the analysis of the unknown samples. To ensure ongoing accuracy, QCs were analyzed regularly throughout the sequence. The analytical batch concluded with a re-acquisition of the calibration curve.

### Method optimization

#### Selection of chromatographic conditions

To optimize chromatographic separation and peak resolution, standard solutions of the analytes at various concentrations in pooled urine were analyzed using different mobile phase compositions and gradient ratios. Specifically, 0.1% aqueous formic acid was tested in combination with varying ratios of acetonitrile and methanol, each containing 0.1% formic acid.

#### Online SPE clean-up and enrichment step

For the online SPE enrichment and clean-up step, PLRP-S, Strata-X, and X-Bridge C18 cartridges were tested by analyzing the QCM. The performance of each cartridge was evaluated by comparing the response of each analyte in terms of signal to noise ratio (S/N), peak width at 5% height, and peak resolution (Rs). After selecting the optimal cartridge, the loading conditions were refined by testing different percentages of methanol (from 5 to 40%, with incremental steps of 5%) and different injection volumes (from 20 to 100 µL, with incremental steps of 20 µL).

### Method validation

The method was validated according to the ICH M10 guideline on bioanalytical method validation and study sample analysis, evaluating sensitivity, precision, accuracy, and matrix effect [35]. Additionally, dilution integrity, long-term stability, selectivity, and extraction recovery were assessed. Due to its later addition to the method, 7-oxo-MINCH was subjected to a partial validation.

#### Enzymatic hydrolysis

The enzymatic hydrolysis of the glucuronide conjugates of plasticizer metabolites was achieved using β-glucuronidase from *E. coli*, as previously recommended [30]. Deconjugation efficiency was assessed by monitoring the hydrolysis of 4-MUG into 4-methylumbelliferone (4-MU, CAS 90-33-5), which served as a marker for complete reaction [36]. To follow this reaction, the 4-MU and 4-MU-^13^C_4_ MS/MS transitions were monitored (ions m/z 175 → 133 and 179 → 135, respectively). To this end, pooled urine samples were fortified with 4-MUG at six concentration levels (6.25, 12.5, 25, 50, 100, 500 µg/L), spiked with 40 µL of 4-MU-^13^C_4_ (500 µg/L in water) as internal standard and added with 40 µL of enzyme solution. These samples were kept at 37 °C for varying durations (0, 30, 60, 90, 120, 180, 240 min). The enzymatic activity was quenched with aqueous acetic acid, and the samples were processed according to the analytical protocol. Hydrolysis efficiency was evaluated by monitoring the formation of 4-MU across all concentration levels and incubation times. The deconjugation process was considered complete when the 4-MU signal reached a stable plateau. Moreover, the effectiveness of aqueous acetic acid as a quenching agent was verified; for this purpose, three pooled urine samples (fortified with 500 µg/L of 4-MUG and 50 µg/L of 4-MU-^13^C_4_) were treated with aqueous acetic acid and then incubated with the enzyme at 37 °C for 60 min.

Finally, to determine the optimal hydrolysis time and the proportion of glucuronide conjugates in real samples, urine from six volunteers (3 males and 3 females) was analyzed. These samples were subjected to hydrolysis for varying intervals (from 0 to 2 h), and the concentrations of free and total metabolites were compared.

#### Selectivity, carryover effect, and IS-interference

To assess selectivity, solvent blanks (ultrapure water), reagent blanks (a mixture containing all the chemical reagents and solvents used in the method, but without the matrix), procedural blanks (ultrapure water processed through the entire analytical workflow), and matrix blanks (unspiked urine processed through the entire analytical workflow) were analyzed.

Potential carryover was investigated by monitoring solvent blanks injected after the most concentrated calibrator and samples with analyte concentrations exceeding the upper limit of quantification (ULOQ).

Additionally, potential interference from the internal standards to the analyte was evaluated by analyzing matrix blanks both with and without the internal standards.

Except for cases where analyte-free matrices were unavailable, the cumulative response of any interfering peaks eluting within a narrow retention time window was calculated; selectivity was confirmed as this sum remained below 20% of the lower limit of quantification (LLOQ) signal. The same threshold was applied to define a negligible carryover effect.

Selectivity was also assessed for the internal standards, applying a stricter acceptance criterion of 5% of their mean peak area [35].

#### Calibration curve and limit of quantification

Calibration curves consisted of at least six non-zero points and a blank; the function was generated by plotting the area ratios (*y*) against the nominal concentrations (*x*, µg/L), applying a 1/*x* weighted least-squares linear regression model. The linear dynamic range, spanning from the LLOQ to the ULOQ, was established by accounting for potential deviations from linearity caused by signal saturation.

LLOQ was determined by analyzing three independent sets of calibration standard solutions across different analytical sequences. For each set, the LLOQ was calculated as $10\bullet \mathrm{S}\mathrm{E}\mathrm{q}/m$ (where SEq is the standard error of intercept, and *m* is the slope of the calibration curve) [37, 38]; these estimations were averaged to provide a preliminary calculated LLOQ (Supplementary Table 3), which was then experimentally verified by analyzing five independent replicates of spiked pooled urine at concentration levels surrounding the estimate. The lowest spiked concentration for which precision (RSD ≤ 20%) and accuracy (≥ 80% and ≤ 120%) remained within the limits established by international guidelines [35], was adopted as the LLOQ.

#### Extraction recovery

To evaluate the extraction efficiency of the method, three aqueous spikes at three concentration levels were analyzed using two distinct LC configurations [25]: an integrated online SPE-LC chromatographic run (a) and a chromatographic run bypassing the online SPE system (b). Extraction recovery was determined by comparing the peak areas, as follows: $\% \mathrm{R}\mathrm{e}\mathrm{c}={\mathrm{A}\mathrm{r}\mathrm{e}\mathrm{a}}_{a}/{\mathrm{A}\mathrm{r}\mathrm{e}\mathrm{a}}_{b}\cdot 100$. The test was performed adopting a reduced injection volume of 20 μL to prevent column overloading.

#### Precision, accuracy, and long-term stability

To evaluate precision and accuracy, five replicates at each QC level were analyzed across three independent analytical batches. Each batch was processed on different days using freshly prepared QC and calibration standards.

Intra-run precision was expressed for each QC level as the mean of the relative standard deviation % RDS values calculated within each of the three analytical sequences.

Between-run precision was reported as the % RSD of all fifteen replicates per QC level, while inter-run accuracy was calculated as the percentage recovery of the mean measured concentration relative to the theoretical value [35].

Analyte stability in the urine matrix was evaluated using a leftover QCL aliquot stored at −20 °C for six months. This duration was established to cover the standard turnaround time for sample processing and to accommodate any potential delays. To comply with analytical guidelines, the sample underwent three freeze-thaw cycles [35]. Under standard protocols, urine samples are thawed once for analysis and potentially a second time for verification; the inclusion of a third cycle extends the stability assessment to cover extreme handling conditions, such as accidental freezer failure. Stability samples were prepared in triplicate and quantified against a calibration curve constructed using freshly prepared standards. Stability was evaluated as % accuracy; each analyte was considered stable when its concentration was within ± 15% of its nominal concentration.

#### Matrix effect and dilution integrity

Inter-individual matrix effect was investigated by analyzing independent calibration sets prepared in six different individual urine lots (from 3 males and 3 females). The % RSD of the slopes was then calculated. In accordance with validation criteria, a significant matrix effect was ruled out for % RSD values ≤ 5 [39]. For % RSD values greater than 5 and less than or equal to 10, the impact of inter-individual matrix effect on quantification was considered of minor relevance.

This evaluation subsequently focused on three calibration levels (levels 1, 2, and 3, which were analyte-specific) to thoroughly assess the inter-individual variability across the entire calibration range. By back-calculating the blank-subtracted concentrations against the pooled urine calibration curve, inter-matrix precision % RSD and accuracy were determined. This approach allowed us to quantify the potential bias introduced by the specific matrix composition of individual samples compared to the pooled reference.

To handle samples exceeding the ULOQ for MEP, 5-cx-MEPTP, and MnBP, a pre-analytical dilution with ultrapure water was implemented. The impact of this procedure on accuracy was evaluated, with an acceptance threshold for bias established at ± 15%.

### External verification

The method’s performance was externally verified through participation in the German External Quality Assessment Scheme (G-EQUAS) [40]; the evaluation spanned rounds 66 to 75 (excluding round 73). Over this period, G-EQUAS progressively expanded its panel of substances; consequently, a comprehensive evaluation of most analytes (5-OH-MEHP, 5-oxo-MEHP, 5-cx-MEPP, MEHP, MnBP, MiBP, MBzP, MEP, OH-MiNP, cx-MiNP, MnHxP, 7-OH-MINCH, 5-OH-MEHTP, 5-cx-MEPTP) became possible starting from round 74. Each round involved the analysis of two distinct urine samples containing blind concentrations, representing low and high levels, specifically designed to reflect the range of exposure typically encountered in the general population.

### Method application

The method was applied to 46 urine samples collected from adults belonging to the Italian general population. These were residual clinical specimens obtained from the hospital’s clinical chemistry department after routine diagnostic testing. In accordance with institutional regulations, the use of anonymized residual samples is permitted for method development, optimization, and validation purposes. To ensure stability, all samples were stored at −20 °C until the time of analysis. These results were compared with those of other European studies, as reported by the HBM4EU dashboard.

### Statistics

The analysis of data for method validation and the descriptive statistics of plasticizer metabolites in urine samples were performed with Microsoft Excel.

## Results and discussion

### Method optimization

#### Chromatography

Based on the evaluation of chromatograms under various SPE and LC conditions, the final elution parameters (eluents, flow rates, and timing) were defined as detailed in Table 4. Acetonitrile containing 0.1% formic acid was selected as the optimal organic modifier, as it provided superior peak resolution and sharper peaks, compared to methanol-based mixtures. Although methanol was tested to enhance ᴨ-ᴨ interactions between the aromatic analytes and phenyl-hexyl stationary phase [41], theoretically improving selectivity for isomeric compounds, it resulted in poor chromatographic performance. Specifically, peak tailing, retention times, and system backpressure increased proportionally with the methanol fraction in mobile phase B (data not shown). Given the straightforward development achieved with acetonitrile, further optimization with methanol was deemed unnecessary and was not pursued.

**Fig. 1:**
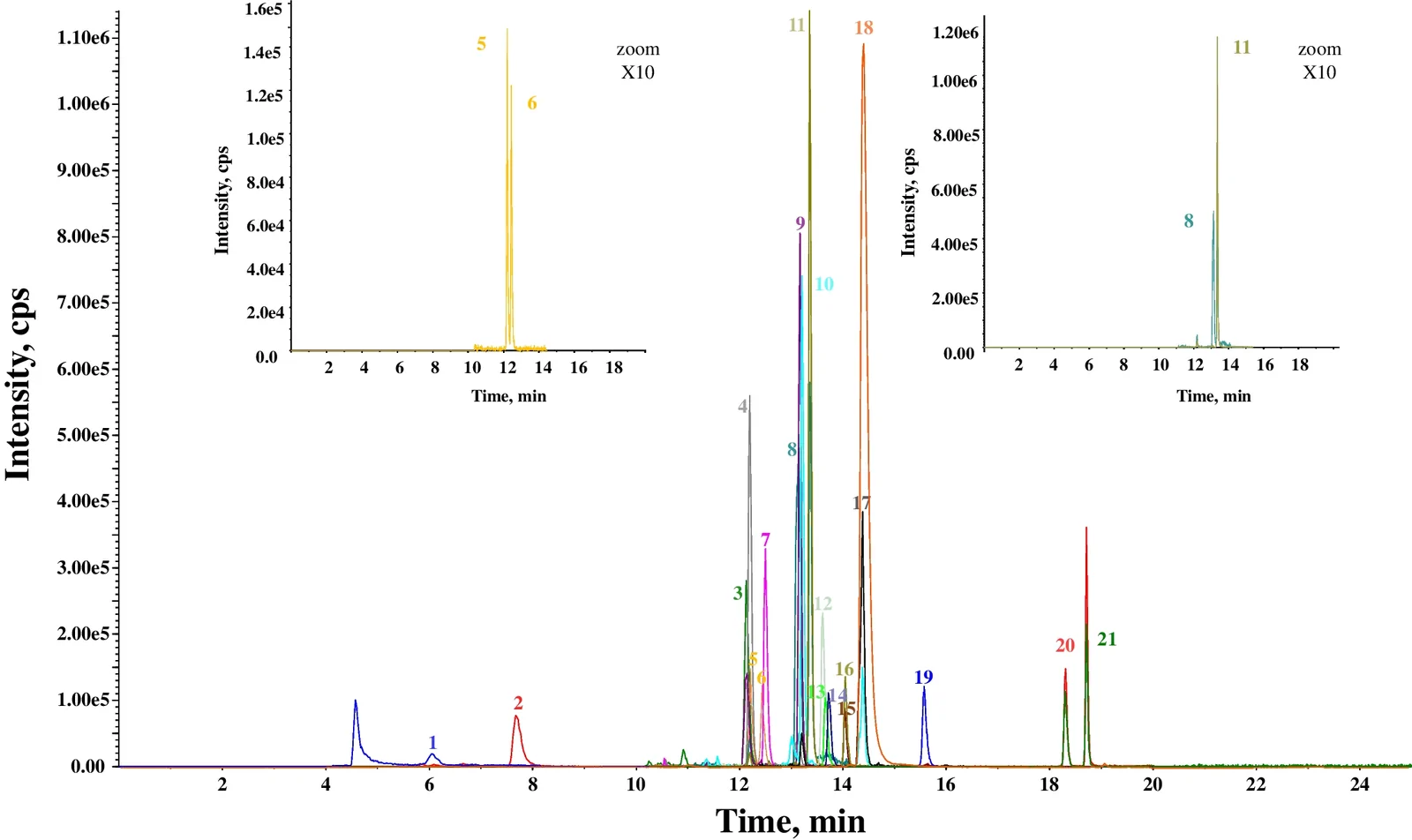
Superimposed extracted ion chromatogram of a non-hydrolyzed spiked pooled urine with each analyte at concentrations of 6.5 μg/L. **1**, MMP; **2**, MEP; **3**, 5-OH-MEHP; **4**, 5-cx-MEPP; **5**, MiBP; **6**, MnBP; **7**, 5-oxo-MEHP; **8**, OH-MiNP; **9**, 5-OH-MEHTP; **10**, cx-MiNP; **11**, 5-cx-MEPTP; **12**, 7-OH-MINCH; **13**, MBzP; **14**, MCHP; **15**, cx-MiDP; **16**, MnPP; **17**, OH-MPHP; **18**, 7-oxo-MINCH; **19**, MnHxP; **20**, MEHP; **21**, MnOP

A relevant aspect of the method optimization was the satisfactory separation of several metabolites. Notably, the resolution of positional isomers (5-OH-MEHP and 5-OH-MEHTP, MiBP and MnBP, MEHP and MnOP) and functional isomers (OH-MiNP and 5-cx-MEPTP, cx-MiNP and OH-MPHP) was achieved by introducing a 3.5-min isocratic step (10.5 to 14.0 min) at 55% aqueous phase (A_b_). Figure 1 shows the chromatogram resulting from the analysis of a pooled urine calibration standard with 6.5 μg/L of each analyte. The enlarged panels demonstrate that adequate discrimination between MiBP and MnBP (left panel) and between OH-MiNP and 5-cx-MEPTP (right panel) was achieved, with peak resolutions of 1.4 (sub-optimal) and 1.6 (optimal), respectively.

**Fig. 2:**
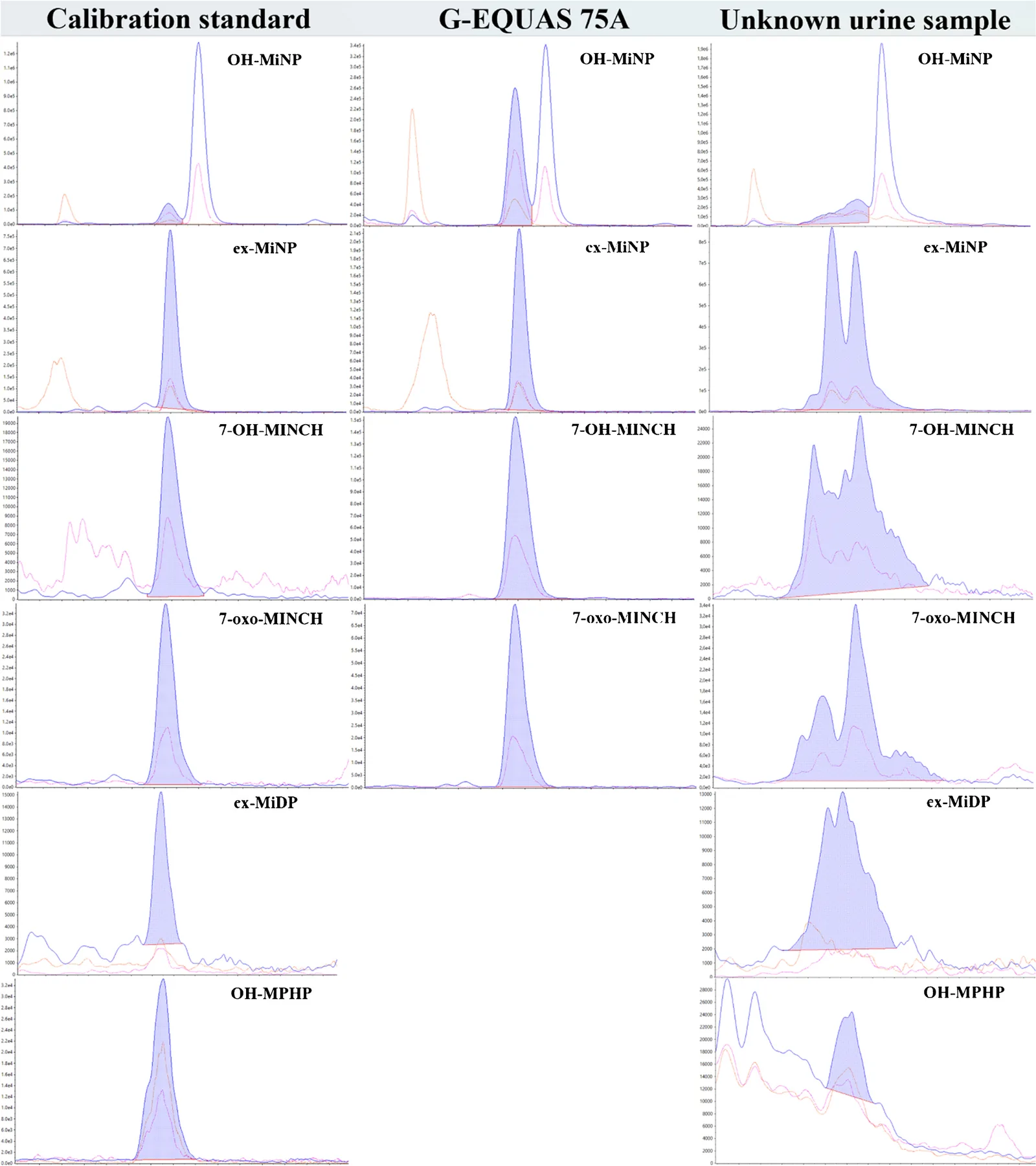
Comparison of superimposed extracted ion chromatograms of OH-MiNP, cx-MiNP, 7-OH-MINCH, 7-oxo-MINCH, cx-MiDP, and OH-MPHP obtained from the pure standards in the calibration solution (left column), the analytes in the G-EQUAS 75 A sample (middle column), and a hydrolyzed urine sample from a subject of the general population (right column). The analyte peak is highlighted by shading

The analysis of urinary plasticizer metabolites revealed additional complexities. Specifically, six analytes, OH-MiNP, cx-MiNP (both DiNP metabolites), 7-OH-MINCH, 7-oxo-MINCH (both DINCH metabolites), cx-MiDP (DiDP metabolite), and OH-MPHP (DPHP metabolite), exhibited chromatographic profiles in urine after hydrolysis that differed from those observed in spiked urine and in G-EQUAS control material (Fig. 2). As previously reported [29, 30], the occurrence of multiple peaks in authentic specimens indicates the presence of various plasticizer isomers (DiNP, DINCH, and DiDP). This pattern reflects exposure to commercial plasticizer mixtures rather than single, pure compounds. Consequently, following the literature, these metabolites were quantified by integrating the total signal of the isomeric cluster eluting within the retention time window of the corresponding pure standards, as reported in Fig. 2 [23, 30, 42]. DPHP is derived from an isomeric mixture of alcohols where 2-propyl-heptanol is the main component, supplemented by minor isomers (2-propyl-4-methylhexanol and 2-propyl-5-methylhexanol). However, the main analytical challenge lies in the determination of DPHP metabolites, due to their co-elution with the trailing edge of DiDP metabolites [31].

For these six analytes, peak identification was verified through ion ratio monitoring; values were required to deviate no more than ± 25% from the reference mean obtained from the calibration standards [43]. To validate the reliability of this integration strategy, repeatability was assessed through independent analytical replicates. Specifically, two aliquots of urine samples were processed and analyzed, and the % RSD of duplicate measurements was calculated, according to Åsberg and Bolann [44].

The calculated % RSD values were 7.0% for OH-MiNP (*n* = 16 pairs), 3.4% for cx-MiNP (*n* = 28), 5.6% for 7-OH-MINCH (*n* = 11), 13.7% for 7-oxo-MINCH (*n* = 4), and 6.8% for OH-MPHP (*n* = 6). Regarding cx-MiDP, since only one pair of measurements was ≥ LLOQ, the imprecision was expressed as the absolute difference in percentage of the mean, which yielded a value of 3.7%. All repeatability results fell within the 15% limit established by the validation guidelines [35], thus confirming methodological compliance.

#### Online SPE clean-up and enrichment step

Among the tested options, the Strata-X cartridge exhibited the poorest performance characterized by the lowest S/N ratios, the broadest peak widths (at 5% height), and consequently, the lowest peak resolution. X-Bridge and PLRP-S cartridges showed comparable results for most analytes; however, the X-Bridge failed to retain MMP. Performance data from the preliminary screening of SPE cartridges are reported in the Supplementary Material (Figures S1, S2 and S3).

Regarding the loading phase, increasing the methanol content up to 20% significantly enhanced the S/N ratio, particularly for MMP and MEP; however, higher concentrations caused premature analyte breakthrough. Consequently, a loading solution with 20% methanol and an injection volume of 100 µL were adopted to maximize sensitivity for all target analytes without compromising selectivity and retention efficiency.

### Method validation

#### Enzymatic hydrolysis

The hydrolysis of 4-MUG proceeded rapidly, reaching completion by the first check (0 h of incubation, Supplementary Figure 4). The absence of 4-MU in the acidified samples confirmed that the addition of aqueous acetic acid to lower the pH to 4.0 effectively quenched the enzymatic activity.

In the experiments conducted on urine samples, most analytes showed constant signal across all incubation times, indicating that hydrolysis was virtually instantaneous upon enzyme addition. The only exception was 5-OH-MEHP, which exhibited an increasing signal up to 30 min before reaching a plateau (Supplementary Figure 5). Based on these findings, an incubation time of 1 h was established to ensure exhaustive hydrolysis for all target analytes. Post-incubation acidification was performed to stabilize the matrix and inhibit salt precipitation, maintaining sample integrity during autosampler residence time.

The degree of glucuronidation of plasticizer metabolites in the urine of six individuals showed significant variation across different chemicals (Table 3 and Supplementary Figure 6). For nine metabolites, the degree of conjugation was nearly complete: 5-OH-MEHP (96.9 ± 0.9%), MiBP (94.5 ± 2.9%), MnBP (95.9 ± 1.7%), 5-oxo-MEHP (96.1 ± 1.6%), OH-MiNP (91.1 ± 7.3%), 7-OH-MINCH (100%), MBzP (97.5 ± 0.8%), 7-oxo-MINCH (98.1 ± 1.6%), and MEHP (88.3 ± 8.3%). In contrast, for five other metabolites, the glucuronidation degree ranged from 7 to 59%: MEP (28.2 ± 9.2%), 5-cx-MEPP (48.6 ± 12.9%), 5-OH-MEHTP (59.3 ± 33.0%), cx-MiNP (39.0 ± 12.2%), and 5-cx-MEPTP (7.0 ± 5.7%). For the remaining metabolites, concentrations below the LLOQ precluded the assessment of the glucuronidation degree. In general, a lower degree of conjugation was observed for small metabolites (MEP from DEP), for those bearing a carboxyl group, and for the metabolites of DEHTP [45].

**Table 5:** Assay validation: linearity range (from LLOQ to ULOQ), precision (% RSD) and % accuracy at LLOQ, coefficient of determination (*R*^2^) of the calibration curves, intra-run precision (% RSD), between-run concentration, between-run precision (% RSD), and between-run % accuracy for QCL, QCM, and QCH. In the last column, the 6-month stability of QCL, expressed as % accuracy vs the nominal concentration, is reported

Analyte	LLOQ–ULOQ (µg/L)	LLOQ precision % RSD *n* = 5	LLOQ % accuracy *n* = 5	*R*^2^	QC	Level (µg/L)	Intra-run precision % RSD (Min–Max) *n* = 3	Between-run concentration ± Std Dev (µg/L) *n* = 15	Between-run precision % RSD *n* = 15	Between-run Mean % accuracy ± Std Dev (Min–Max) *n* = 15	Six-month stability % accuracy *n* = 3
MMP	0.78–50.00	12.6	89.2	0.9980	QCL	1.60	3.6 (2.1–4.6)	1.69 ± 0.10	6.1	105.4 ± 6.4 (94.7–120.9)	93
					QCM	5.00	3.1 (1.5–5.4)	5.21 ± 0.21	4.0	104.1 ± 4.2 (95.0–109.9)	
					QCH	40.00	1.8 (1.1–2.8)	40.26 ± 1.09	2.7	100.6 ± 2.7 (97.1–106.2)	
MEP	0.78–50.00	6.0	115.2	0.9990	QCL	1.60	4.0 (2.4–6.0)	1.68 ± 0.07	4.2	105.1 ± 4.4 (97.0–113.7)	94
					QCM	5.00	2.9 (2.2–3.7)	5.22 ± 0.25	4.8	104.4 ± 5.0 (93.4–110.7)	
					QCH	40.00	2.1 (1.8–2.6)	40.14 ± 0.95	2.4	100.3 ± 2.4 (97.0–104.3)	
5-OH-MEHP	0.39–50.00	7.9	111.4	0.9976	QCL	0.80	2.5 (1.9–2.9)	0.84 ± 0.03	3.3	105.0 ± 3.4 (98.3–110.6)	96
					QCM	5.00	3.0 (2.2–3.8)	4.99 ± 0.19	3.8	99.9 ± 3.8 (91.2–105.4)	
					QCH	40.00	1.7 (1.3–2.2)	40.03 ± 0.80	2.0	100.1 ± 2.0 (97.6–104.2)	
5-cx-MEPP	0.20–25.00	6.3	101.1	0.9965	QCL	0.40	4.5 (3.6–6.4)	0.39 ± 0.02	5.4	98.3 ± 5.3 (90.2–108.8)	93
					QCM	3.20	4.8 (2.8–6.8)	3.40 ± 0.19	5.6	102.3 ± 6.1 (93.1–112.1)	
					QCH	20.00	3.6 (3.0–4.5)	20.53 ± 1.08	5.3	102.6 ± 5.4 (92.5–113.2)	
MiBP	0.78–50.00	12.5	101.2	0.9989	QCL	1.60	4.9 (3.7–6.2)	1.66 ± 0.09	5.3	103.6 ± 5.5 (93.8–111.2)	89
					QCM	5.00	3.9 (2.5–5.9)	5.25 ± 0.21	3.9	105.1 ± 4.1 (100.2–114.4)	
					QCH	40.00	3.8 (2.4–5.0)	41.08 ± 1.59	3.9	102.7 ± 4.0 (96.0–110.6)	
MnBP	0.78–50.00	17.9	117.0	0.9988	QCL	1.60	13.8 (6.0–23.3)	1.65 ± 0.26	15.6	102.9 ± 16.0 (74.5–123.9)	94
					QCM	5.00	6.6 (3.6–10.6)	5.16 ± 0.36	7.1	103.2 ± 7.3 (84.8–114.5)	
					QCH	40.00	2.1 (1.8–2.5)	40.47 ± 0.81	2.0	101.2 ± 2.0 (98.2–104.7)	
5-oxo MEHP	0.10–25.00	4.5	103.4	0.9989	QCL	0.20	2.9 (1.3–4.0)	0.21 ± 0.01	5.7	104.2 ± 6.0 (91.8–114.0)	100
					QCM	1.60	1.8 (1.3–2.1)	1.70 ± 0.05	2.8	106.0 ± 2.9 (102.2–111.4)	
					QCH	20.00	2.4 (2.0–2.7)	20.30 ± 0.59	2.9	101.5 ± 3.0 (96.3–107.4)	
OH-MiNP	0.20–25.00	6.0	93.8	0.9895	QCL	0.40	3.9 (1.9–5.3)	0.39 ± 0.02	4.0	96.5 ± 3.9 (88.5–102.5)	90
					QCM	3.20	3.8 (2.8–5.3)	3.47 ± 0.22	6.3	104.3 ± 6.9 (95.1–120.4)	
					QCH	20.00	3.3 (1.9–6.0)	21.04 ± 1.23	5.9	105.2 ± 6.2 (95.6–115.1)	
5-OH-MEHTP	0.10–25.00	4.7	101.8	0.9981	QCL	0.20	4.6 (3.0–6.3)	0.20 ± 0.01	6.0	100.8 ± 6.1 (92.7–111.0)	93
					QCM	1.60	3.1 (2.5–3.5)	1.68 ± 0.06	3.4	104.9 ± 3.6 (98.6–109.9)	
					QCH	20.00	3.3 (2.0–4.2)	20.03 ± 0.76	3.8	100.1 ± 3.8 (95.8–110.4)	
cx-MiNP	0.10–25.00	9.0	107.2	0.9975	QCL	0.20	8.5 (6.8–10.8)	0.21 ± 0.02	9.3	104.8 ± 9.7 (88.4–117.4)	99
					QCM	1.60	2.9 (1.9–5.0)	1.71 ± 0.08	4.9	107.1 ± 5.2 (102.2–121.7)	
					QCH	20.00	2.0 (0.8–3.0)	20.11 ± 0.51	2.5	100.5 ± 2.5 (94.7–105.0)	
5-cx-MEPTP	0.78–50.00	10.2	101.6	0.9974	QCL	1.60	5.8 (2.3–7.8)	1.69 ± 0.21	12.3	105.9 ± 13.0 (90.2–126.7)	105
					QCM	5.00	5.3 (4.4–6.1)	5.38 ± 0.32	6.0	107.5 ± 6.4 (93.6–117.7)	
					QCH	40.00	2.3 (1.2–2.9)	40.32 ± 2.16	5.4	100.8 ± 5.4 (89.8–107.0)	
7-OH-MINCH	0.39–50.00	3.8	112.8	0.9981	QCL	0.80	3.6 (2.3–4.3)	0.84 ± 0.04	4.7	105.1 ± 4.9 (96.9–116.1)	91
					QCM	5.00	2.7 (2.0–3.2)	4.86 ± 0.15	3.1	97.2 ± 3.1 (92.0–102.3)	
					QCH	40.00	2.7 (1.7–3.6)	41.19 ± 1.52	3.7	103.0 ± 3.8 (98.0–110.3)	
MBzP	0.20–50.00	6.5	109.7	0.9982	QCL	0.40	7.4 (5.3–9.3)	0.40 ± 0.04	9.1	99.3 ± 9.0 (86.1–117.7)	94
					QCM	3.20	4.3 (2.6–6.3)	3.35 ± 0.14	4.3	101.0 ± 4.5 (92.2–108.4)	
					QCH	40.00	3.5 (2.4–4.7)	40.31 ± 1.92	4.8	100.8 ± 4.8 (90.8–109.5)	
MCHP	0.20–50.00	7.8	117.3	0.9986	QCL	0.40	10.8 (3.2–19.0)	0.41 ± 0.05	13.4	102.0 ± 13.7 (69.9–116.0)	86
					QCM	3.20	4.3 (2.7–5.9)	3.35 ± 0.18	5.4	100.8 ± 5.7 (92.5–109.8)	
					QCH	40.00	3.6 (2.9–4.9)	39.60 ± 1.56	3.9	99.0 ± 3.9 (94.1–109.6)	
cx-MiDP	0.39–50.00	2.4	112.9	0.9973	QCL	0.80	8.5 (6.2–11.7)	0.79 ± 0.07	8.4	99.3 ± 8.3 (77.3–112.1)	98
					QCM	5.00	3.4 (2.5–3.8)	5.21 ± 0.35	6.7	104.1 ± 7.0 (93.4–116.0)	
					QCH	40.00	5.3 (3.4–8.6)	38.59 ± 2.38	6.2	96.5 ± 5.9 (79.1–105.8)	
MnPP	0.10–50.00	6.6	110.3	0.9994	QCL	0.20	5.0 (3.2–6.8)	0.20 ± 0.01	5.9	98.3 ± 5.8 (89.1–110.7)	89
					QCM	3.20	2.2 (1.3–3.4)	3.31 ± 0.15	4.5	99.8 ± 4.8 (93.2–107.7)	
					QCH	40.00	2.8 (1.7–4.5)	39.55 ± 2.17	5.5	98.9 ± 5.4 (90.9–105.9)	
OH-MPHP	0.10–50.00	2.8	113.6	0.9986	QCL	0.20	1.8 (1.4–2.2)	0.21 ± 0.01	4.3	105.5 ± 4.6 (98.5–112.5)	91
					QCM	3.20	3.1 (1.7–5.4)	3.49 ± 0.13	3.6	105.1 ± 4.0 (97.7–111.7)	
					QCH	40.00	4.8 (2.9–7.9)	40.31 ± 2.38	5.9	100.8 ± 5.9 (82.9–108.3)	
7-oxo-MINCH	0.16–60.60	N/A	N/A	0.9990	QCL	0.40	1.0	N/A	N/A	105.1*	N/A
					QCM	4.00	1.4	N/A	N/A	108.5*	
					QCH	40.00	3.3	N/A	N/A	107.8*	
MnHxP	0.10–50.00	2.5	118.7	0.9997	QCL	0.20	4.1 (3.6–4.9)	0.21 ± 0.01	5.7	103.8 ± 5.9 (92.6–115.9)	89
					QCM	3.20	2.8 (2.4–3.4)	3.42 ± 0.09	2.8	103.0 ± 2.9 (97.5–108.2)	
					QCH	40.00	2.5 (1.8–3.0)	40.94 ± 1.01	2.5	102.3 ± 2.5 (98.9–107.8)	
MEHP	0.39–50.00	11.8	95.8	0.9989	QCL	0.80	2.9 (1.8–4.1)	0.84 ± 0.06	6.7	105.6 ± 7.1 (94.2–115.3)	96
					QCM	5.00	2.4 (2.2–2.7)	5.20 ± 0.17	3.3	103.9 ± 3.5 (97.0–109.5)	
					QCH	40.00	3.0 (2.3–4.1)	40.42 ± 1.15	2.9	101.1 ± 2.9 (94.2–104.9)	
MnOP	0.10–50.00	4.0	111.5	0.9989	QCL	0.20	3.7 (1.5–7.2)	0.20 ± 0.01	5.8	100.0 ± 5.8 (87.9–107.5)	85
					QCM	3.20	3.5 (2.0–5.7)	3.43 ± 0.20	5.7	103.6 ± 6.2 (95.7–115.9)	
					QCH	40.00	2.3 (1.2–2.9)	40.59 ± 0.98	2.4	101.5 ± 2.4 (97.9–106.2)	

*From intra-run test

*N/A* data not available

#### Selectivity, carryover effect, and IS-interference

The implementation of an online SPE system required specific measures to mitigate background interference, as the loading step promotes the pre-concentration of system contaminants. While a C18 column placed between the analytical pump and the injector provided sufficient delay of interferences, it was inadequate for retaining contaminants (e.g., MMP) upstream of the SPE system. This issue was resolved by employing a pentafluorophenyl stationary phase (Hypersil GOLD PFP), which effectively shifted background signals away from the analyte retention windows.

Carryover effect, reagents, and the overall procedure did not introduce significant interfering signals; however, the urinary matrix itself exhibited quantifiable levels of MMP, MEP, 5-OH-MEHP, 5-cx-MEPP, MiBP, MnBP, 5-OH-MEHTP, cx-MiNP, and 5-cx-MEPTP, even in the absence of β-glucuronidase treatment (Supplementary Tables 2 and 5).

The concentration of the internal standards in the MIX IS PHTs was optimized to ensure the absence of signals of the non-deuterated analogs (Supplementary Table 1).

#### Calibration curve, limits of quantification, precision, accuracy, long-term stability, and extraction recovery

Validation parameters are summarized in Table 5 and in Supplementary Figures 7–10. Calibration curves exhibited good linearity, with coefficients of determination (*R*^2^) exceeding 0.9895 for all analytes. LLOQs precision and accuracy ranged from 2.4% (cx-MiDP) to 17.9% (MnBP) and from 89.2% (MMP) to 118.7% (MnHxP), respectively. Intra-run precision ranged from 1.0% (7-oxo-MINCH at QCL) to 13.8% (MnBP at QCL), while between-run precision varied between 2.0% (5-OH-MEHP and MnBP at QCH) and 15.6% (MnBP at QCL). Between-run accuracy was within the 96.5–107.5% range across all analytes and QC levels. The method demonstrated full compliance with ICH M10 standards, even at LLOQ level, as both precision and accuracy values remained ≤ 15% (≤ 20% for LLOQ) and between 85 and 115% (80–120% for LLOQ), respectively.

The 6-month stability study conducted on QCL replicates confirmed the integrity of all target analytes under storage conditions, with accuracy ranging from 85% (MnOP) to 105% (5-cx-MEPTP).

Recovery data, detailed in Supplementary Table 4 and Supplementary Figure 11, were highly satisfactory, between 90 and 112%; these results confirm the suitability of the online SPE method for the efficient extraction of the analytes [46].

#### Matrix effect

The matrix effect was evaluated by assessing the relative standard deviation of the slopes (slope % RSD) deriving from calibration curves, each prepared in individual urine from six volunteers; the results are presented in Table 3 and in Supplementary Figures 12–13. This parameter reflects the inter-individual variability; for most of the analytes (14 out of 21), the slope % RSD was ≤ 5%, indicating a negligible matrix effect [47]. For the remaining analytes, the slope % RSD ranged from 6.1 to 9.3% (5-OH-MEHP, MnBP, 5-oxo-MEHP, 5-cx-MEPTP, and MCHP), while values of 13.9 and 13.6 were obtained for MBzP and cx-MiDP, respectively.

Focusing on the three distinct calibration levels, although a higher degree of variability is inherently expected at lower concentrations, this pattern must be contextualized for those metabolites showing detectable levels in blank matrices (Supplementary Table 5). For this assessment, we performed a matrix-specific blank subtraction by deducting the concentration found in each non-spiked individual urine from the corresponding calibration solution. Endogenous levels of MMP, MEP, MnBP, 5-OH-MEHTP, cx-MiNP, and 5-cx-MEPTP in the blank matrices may have potentially affected the discrimination of the spiked amount at the lowest concentration levels. Regarding MBzP and cx-MiDP, while they showed higher slope % RSD, the inter-individual precision at levels 2 and 3 never exceeded 16%.

The overall inter-individual accuracy for the analytes remained highly satisfactory, ranging from 86 to 111%, with the only exception of MnBP (84% at level 1); however, this marginal non-compliance may be attributable to the bias introduced by subtraction of urinary background levels.

The sample dilution introduced a minimal bias (ranging from 4 to 6%), which did not significantly impact the quantification of MEP, 5-cx-MEPTP and MnBP.

The overall findings demonstrate that the matrix effect was effectively managed.

#### External verification

Over the years, participation in the G-EQUAS external verification schemes has yielded consistently successful results. During rounds 74 and 75, nearly all available analytes were evaluated, with the exception of 7‑cx‑MINCH, which is not included in the present method (Supplementary Figure 14). These outcomes further confirm the robustness of the developed method. It should be noted, however, that the G-EQUAS control material is prepared by spiking urine with pure analytical standards. This simplifies analyte quantification compared with real urine samples, in which, for a few plasticizers, multiple peaks arising from isomeric metabolites are typically present (Fig. 2).

### Analysis of real urine samples

The concentrations of plasticizer metabolites measured in urine samples from 46 Italian adults are summarized in Table 6; for 7‑oxo‑MINCH, the analysis was performed on a subset of 16 samples.

**Table 6:** Concentration of urinary metabolites of plasticizers in 46 Italian individuals and results from the HBM4EU project across European countries

Parent compound	Analyte	Descriptive statistics of our samples				HBM4EU 2015–2021 median ranges across European countries		
		No. of samples ≥ LLOQ (%)	5th percentile (µg/L)	Median (µg/L)	95th percentile (µg/L)	Minimum median value (µg/L) (country and numerosity)	Maximum median value (μg/L) (country and numerosity)	LLOQ ranges (min–max) (µg/L)
DMP	MMP	39 (85%)	< 0.78	2.09	7.12	1.86 (Denmark, *n* = 100)	6.30 (Germany, *n* = 2257)	0.27–1.00
DEP	MEP	46 (100%)	12.07	38.44	1192.75	8.95 (Hungary, *n* = 262)	79.08 (Spain, *n* = 300)	0.15–1.00
DiBP	MiBP	46 (100%)	2.92	8.29	24.49	11.97 (Czech Republic, *n* = 399)	33.28 (Greece, *n* = 161)	0.16–2.17
DnBP	MnBP	46 (100%)	2.84	8.34	38.20	10.00 (Austria, *n* = 85)	91.80 (Slovakia, *n* = 287)	0.17–3.04
BBzP	MBzP	44 (96%)	0.26	1.33	5.88	1.26 (Slovakia, *n* = 287)	3.53 (Slovenia, *n* = 96)	0.03–2.37
DnPP	MnPP	0 (0%)	< 0.10	< 0.10	< 0.10	< LLOQ^a^		0.04–0.74
DnHxP	MnHxP	8 (17%)	< 0.10	< 0.10	0.38	0.07 (Denmark, *n* = 100)**		0.04
DCHP	MCHP	0 (0%)	< 0.20	< 0.20	< 0.20	< LLOQ^b^		0.15–0.68
DNOP	MnOP	0 (0%)	< 0.10	< 0.10	< 0.10	< LLOQ^b^		0.03–1.50
DEHP	MEHP	42 (91%)	< 0.39	0.93	2.94	0.09 (Austria, *n* = 85)	3.54 (Slovakia, *n* = 287)	0.05–0.61
	5-OH-MEHP	46 (100%)	1.18	3.45	13.18	4.69 (Czech Republic, *n* = 399)	33.70 (Slovakia, *n* = 287)	0.05–2.84
	5-cx-MEPP	46 (100%)	0.77	2.51	5.82	6.63 (Denmark, *n* = 300)	18.38 (Slovenia, *n* = 149)	0.07–0.50
	5-oxo-MEHP	46 (100%)	0.46	1.51	5.00	2.79 (Czech Republic, *n* = 399)	10.81 (Slovenia, *n* = 149)	0.06–2.71
DiNP	OH-MiNP	45 (98%)	0.51	2.56	12.20	1.90 (Belgium, *n* = 133)	16.20 (Slovakia, *n* = 287)	0.03–0.70
	cx-MiNP	46 (100%)	1.04	2.89	13.00	0.28 (Belgium, *n* = 133)	6.89 (Slovenia, *n* = 149)	0.04–0.40
DPHP	OH-MPHP	28 (61%)	< 0.10	0.12	0.52	0.40 (Denmark, *n* = 100)	2.78 (Greece, *n* = 161)	0.02–0.60
DiDP	cx-MiDP	18 (39%)	< 0.39	< 0.39	1.80	0.35 (Denmark, *n* = 100)	1.11 (Belgium, *n* = 300)	0.02–0.37
DEHTP	5-OH-MEHTP	44 (96%)	0.16	0.59	3.26	0.50 (Germany, *n* = 2118)**		0.30
	5-cx-MEPTP	45 (98%)	1.77	11.01	51.00	7.35 (Germany, *n* = 2118)**		0.20
DINCH	7-OH-MINCH	30 (65%)	< 0.39	0.97	7.94	0.70 (Germany, *n* = 60)	3.01 (Denmark, *n* = 300)	0.02–1.00
	7-oxo-MINCH*	6 (38%)	< 0.16	< 0.16	6.09	0.29 (Germany, *n* = 60)	0.95 (Germany, *n* = 2226)	0.05

*Determined in a subset of 16 urine samples

**Only one country provided data

^a^Consistent across Netherlands, Greece, Slovenia, Denmark, Spain, Poland, Hungary, Norway, and Germany

^b^Consistent across Netherlands, Greece, Czech Republic, Slovakia, Slovenia, Denmark, Spain, Poland, Hungary, and Norway

Fifteen metabolites were quantified in more than 60% of the samples; notably, this group included the DINCH metabolite 7-OH-MINCH, as well as DEHTP metabolites. MnHxP, cx‑MiDP, and 7‑oxo‑MINCH were detected in 17–39% of samples, whereas MnPP, MCHP, and MnOP were consistently below the LLOQ. The highest concentrations were observed for MEP, with median and 95th percentile of 38.44 and 1193 µg/L, respectively, followed by 5‑cx‑MEPTP (11.01 and 51.00 µg/L), MnBP (8.34 and 38.20 µg/L), and MiBP (8.29 and 24.49 µg/L). These findings confirm the ubiquitous exposure to DEP, DEHTP, DnBP, and DiBP, as well as widespread occurrence of DINCH. By contrast, the data does not reveal current exposure to DnPP, DCHP, and DNOP. Overall, our data aligns with the European exposure profiles recorded between 2015 and 2021 within the HBM4EU framework (Table 6) [48].

### Methodological considerations

Overall, the present method demonstrates very good performance, although some limitations remain. In particular, the total analysis time is 25 min, which restricts sample throughput. The use of online SPE reduces sample handling and allows for automated preparation, but it requires the addition of a dedicated valve system to the LC-MS/MS apparatus. Furthermore, chromatographic performance could potentially be improved by employing ultra-high performance liquid chromatography. Moreover, the quantification of isomeric metabolites remains analytically challenging, as the co-elution of multiple species can severely complicate peak identification and integration. In this context, high-resolution mass spectrometry (HRMS) enhances selectivity, allowing for the discrimination of functional isomers and filters out isobaric matrix interferences by setting a narrow mass tolerance [29].

The major strength of our method lies in the extensive validation performed using authentic human urine throughout the entire study. While lower LLOQs have been reported elsewhere, it is critical to note that such values are often estimated using synthetic urine [25, 28, 49] or ultrapure water [26]. In contrast, our LLOQs were determined using real pooled urine and subsequently experimentally verified in the same biological matrix. This approach ensures that the reported limits are truly representative of routine analytical performance.

## Conclusion

This work presents a high-throughput analytical method for the determination of 21 urinary metabolites of phthalates, DEHTP, and DINCH. The reliability of the method was rigorously confirmed through internal validation in accordance with international guidelines and further supported by successful participation in the G-EQUAS external quality assessment scheme. A key challenge was the chromatographic co-elution of isomeric mixtures deriving from DiNP, DiDP, and DINCH. This was effectively managed by integrating the cumulative response of the isomeric peaks within defined elution window, using ion ratio monitoring to ensure confident structural identification. The analysis of 46 samples revealed widespread exposure to both regulated plasticizers and phthalate substitutes like DEHTP and DINCH within the general population. The alignment of our findings with European biomonitoring data confirms the applicability of this protocol for large-scale studies.

## Supplementary Information

Below is the link to the electronic supplementary material.

Supplementary file1 (DOCX 272 KB)

## Data Availability

All the relevant data are reported in the paper and/or in the supplementary material.

## Declarations

### Source of biological material

Urine used in the method application was residual clinical specimens obtained from the hospital’s clinical chemistry department after routine diagnostic testing.
