# Publisher Correction: A robust and validated method for the determination of 21 urinary metabolites of 15 plasticizers, including phthalates, DEHTP, and DINCH, by online SPE and liquid chromatography‑tandem mass spectrometry

**DOI:** 10.1007/s00216-026-06709-6

**Published:** 2026-08-03

**Authors:** Rosa Mercadante, Simone Cafagna, Marco Rizzo, Elisa Roggia, Silvia Fustinoni

**Affiliations:** 1 Dipartimento di Scienze Cliniche e di Comunità, Dipartimento di Eccellenza 2023‑2027, Università degli Studi di Milano, Milano, Italy; 2 Toxicology Lab Fondazione IRCCS Ca’ Granda Ospedale Maggiore Policlinico, Milan, Italy

**Publisher Correction: Analytical and Bioanalytical Chemistry (2025) 417:6549–6566**

**https://doi.org/10.1007/s00216-026-06634-8**

The original version of this article contained errors.

Multiple table cross-references have been changed incorrectly throughout the manuscript. These incorrect citations make the paper difficult to follow and may confuse readers.

1. **Materials and Methods**, section "**Standard, calibration, and quality control solutions**"

*"Matrix-matched calibration standards (ranging from 0.10 to 50 µg/L) and quality control (QC) samples at three levels (low, QCL; medium, QCM; and high, QCH; concentrations in Table 3)."*

The reference should be **Table 5**, not **Table 3**, as Table 3 reports the matrix effect and glucuronidation data.

2. **Results and Discussion**, section "**Enzymatic hydrolysis**"

*"The degree of glucuronidation of plasticizer metabolites in the urine of six individuals showed significant variation across different chemicals (Table 5 and Supplementary Figure 6)."*

The reference at the end of the sentence should be **Table 3**, not **Table 5**.

3. **Results and Discussion**, section "**Calibration curve, limits of quantification, precision, accuracy, long-term stability, and extraction recovery**"

*"Validation parameters are summarized in Table 3 and in Supplementary Figures 7–10."*

The correct reference is **Table 5**, not **Table 3**.

4. **Results and Discussion**, section "**Matrix effect**"

*"The matrix effect was evaluated by assessing the relative standard deviation of the slopes (slope % RSD) deriving from calibration curves, each prepared in individual urine from six volunteers; the results are presented in Table 5 and in Supplementary Figures 12–13."*

The correct reference is **Table 3**, not **Table 5**.

In addition, **Table 2 has been altered and is no longer complete**. The table appears to have been truncated, several compounds are duplicated, and an additional header row has been inserted in the middle of the table. These formatting issues significantly affect the readability and accuracy of the table.

Table 2 should have appeared as shown below.

**Figure:**
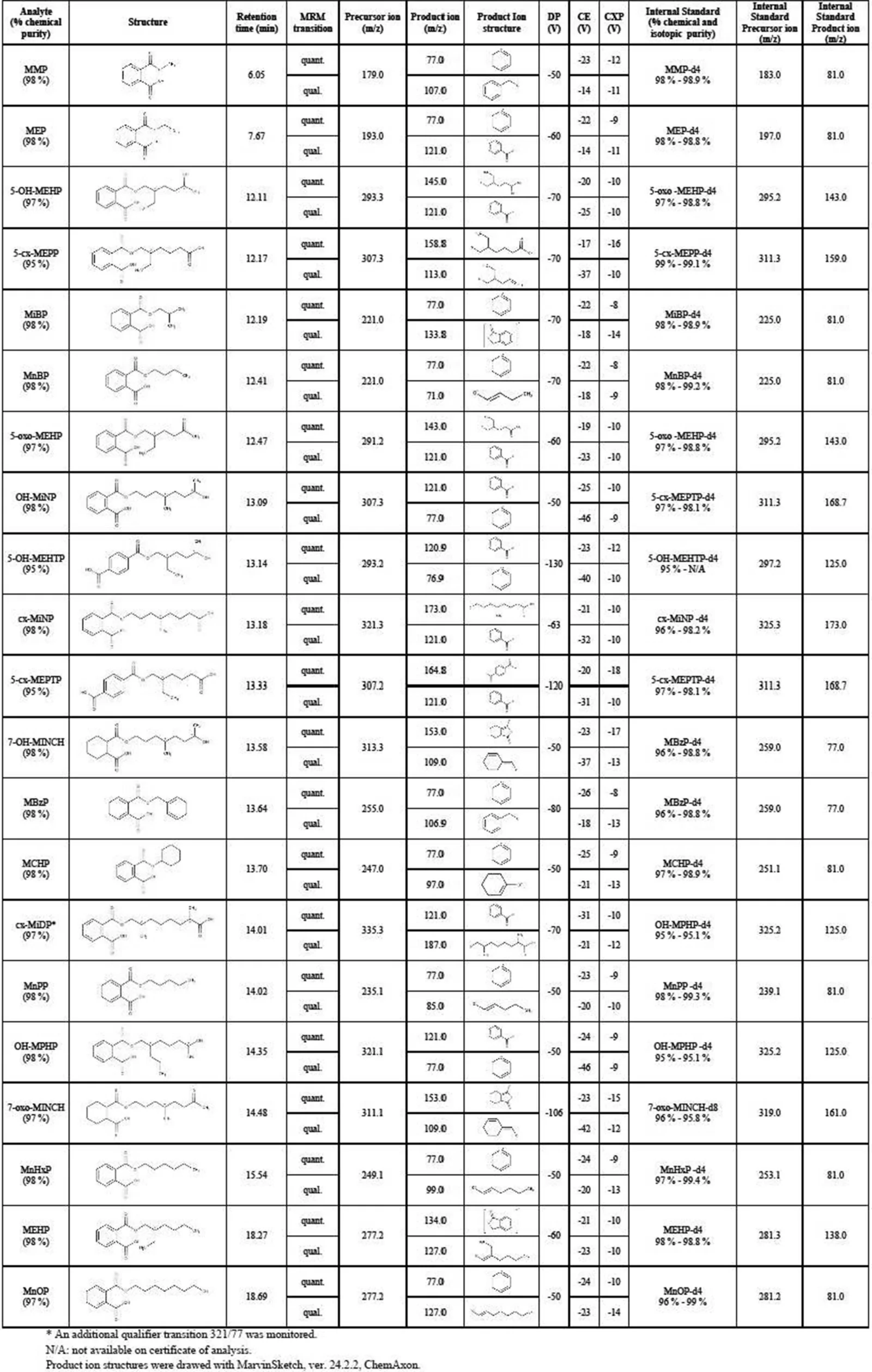

The original article has been corrected.

