# Supplementary material for: A robust and validated method for the determination of 21 urinary metabolites of 15 plasticizers, including phthalates, DEHTP, and DINCH, by online SPE and liquid chromatography-tandem mass spectrometry
Source: Anal Bioanal Chem. 2026 Jul 4;418(19):6285–306. doi: 10.1007/s00216-026-06634-8 (PMC13585993; doi:10.1007/s00216-026-06634-8)
Supplement: Supplementary file 1 [file 216_2026_6634_MOESM1_ESM.docx]

**Supplementary material**

**A robust and validated method for the determination of 21 urinary metabolites of 15 plasticizers, including phthalates, DEHTP and DINCH, by on-line SPE and liquid chromatography tandem mass spectrometry**

Rosa Mercadante ^1#^ and Simone Cafagna ^2#^, Marco Rizzo^1^, Elisa Roggia^1^, Silvia Fustinoni ^1,2§^

^1^Department of Clinical Sciences and Community Health, University of Milan, Milan, Italy

^2^Toxicology lab Fondazione IRCCS Ca' Granda Ospedale Maggiore Policlinico, Milan, Italy

^#^ These authors equally contributed to this work

^§^Author correspondence address:

Via F. Sforza, 35 – 20122 Milan, Italy

phone: +39 02 503 20158

e-mail: silvia.fustinoni@unimi.it

**Keywords:** metabolites of phthalates, terephthalate, DINCH, urine, LC-MS/MS, biological monitoring, general population exposure, method validation

**Supplementary Table 1.** PHTs Internal Standard Mix concentration of each chemical.

| **Internal standard** | **CAS** | **Concentration (µg/L)** |
| --- | --- | --- |
| MMP-d4 | 1276197-40-0 | 451.8 |
| MEP-d4 | 1219806-03-7 | 193.6 |
| 5-oxo -MEHP-d4 | 679789-44-7 | 25.0 |
| 5-cx-MEPP-d4 | 866864-06-4 | 16.2 |
| MiBP-d4 | 1219802-26-2 | 76.5 |
| MnBP-d4 | 478954-81-3 | 114.7 |
| 5-cx-MEPTP-d4 | N/A | 22.6 |
| 5-OH-MEHTP-d4 | N/A | 26.2 |
| cx-MiNP-d4 | 1332966-02-5 | 16.3 |
| MBzP-d4 | 478954-83-5 | 140.4 |
| MCHP-d4 | 1398066-18-6 | 78.9 |
| OH-MPHP-d4 | 1412411-11-0 | 16.9 |
| MnPP-d4 | 1794756-28-7 | 70.2 |
| 7-oxo-MINCH-d8 | N/A | 19.9 |
| MnHxP-d4 | 2469279-12-5 | 48.1 |
| MEHP-d4 | 1276197-22-8 | 46.6 |
| MnOP-d4 | 1398065-74-1 | 61.2 |

N/A = not available as neither provided by the manufacturer nor indexed in major public databases.

**Supplementary Figure 1.** Evaluation of different online SPE stationary phases based on Signal-to-Noise (S/N) ratio. The plot compares Strata-X, X-Bridge, and PLRP-S cartridges across the full panel of plasticizer metabolites. Data points represent the S/N values (log scale) as detailed in the accompanying table. X-Bridge failed to retain MMP.

Comparison of Strata-X, X-Bridge and PLRP-S in terms of S/N Ratio

Log(S/N)

|  | Strata-X | X-Bridge | PLRP-S |
| --- | --- | --- | --- |
| MMP | 45.7 | 0 | 82.8 |
| MEP | 348.2 | 208.2 | 345.9 |
| 5-OH-MEHP | 260.4 | 395.5 | 309.3 |
| 5-cx-MEPP | 432.7 | 502 | 414.8 |
| MiBP | 85.8 | 113.6 | 92.2 |
| MnBP | 142.3 | 125.4 | 146 |
| 5-oxo-MEHP | 1427.6 | 1663.6 | 1615.3 |
| OH-MiNP | 283.1 | 269.3 | 402.4 |
| 5-OH-MEHTP | 1024.9 | 793.2 | 1269.5 |
| cx-MiNP | 1031.8 | 780.1 | 1005.8 |
| 5-cx-MEPTP | 951.7 | 875.2 | 713.4 |
| 7-OH-MINCH | 912.6 | 804.6 | 884.1 |
| MBzP | 904.9 | 1632.6 | 1608.6 |
| MCHP | 553.8 | 921.1 | 878.9 |
| cx-MiDP | 422.3 | 552.9 | 589.1 |
| MnPP | 462.6 | 1658.6 | 1451.8 |
| OH-MPHP | 416.6 | 384.7 | 479 |
| MnHxP | 841.6 | 860.7 | 957.5 |
| MEHP | 539.3 | 326.8 | 457.4 |
| MnOP | 489.8 | 386 | 579.8 |

**Supplementary Figure 2.** Evaluation of different online SPE stationary phases based on peak width (minutes at 5% height). The plot compares Strata-X, X-Bridge, and PLRP-S cartridges across the full panel of plasticizer metabolites. Data points represent the peak width values as detailed in the accompanying table. X-Bridge failed to retain MMP.

Comparison of Strata-X, X-Bridge and PLRP-S in terms of width (measured in minutes at 5% height)

width (measured in minutes at 5% height)

|  | Strata-X | X-Bridge | PLRP-S |
| --- | --- | --- | --- |
| MMP | 0.75 | 0 | 0.64 |
| MEP | 0.86 | 0.5 | 0.6 |
| 5-OH-MEHP | 0.32 | 0.22 | 0.25 |
| 5-cx-MEPP | 0.37 | 0.28 | 0.28 |
| MiBP | 0.22 | 0.24 | 0.2 |
| MnBP | 0.31 | 0.26 | 0.26 |
| 5-oxo-MEHP | 0.47 | 0.36 | 0.38 |
| OH-MiNP | 0.25 | 0.21 | 0.24 |
| 5-OH-MEHTP | 0.43 | 0.34 | 0.35 |
| cx-MiNP | 0.27 | 0.21 | 0.23 |
| 5-cx-MEPTP | 0.3 | 0.21 | 0.22 |
| 7-OH-MINCH | 0.3 | 0.27 | 0.29 |
| MBzP | 0.46 | 0.34 | 0.37 |
| MCHP | 0.45 | 0.35 | 0.36 |
| cx-MiDP | 0.4 | 0.33 | 0.34 |
| MnPP | 0.41 | 0.34 | 0.36 |
| OH-MPHP | 0.31 | 0.24 | 0.25 |
| MnHxP | 0.44 | 0.35 | 0.39 |
| MEHP | 0.26 | 0.22 | 0.24 |
| MnOP | 0.32 | 0.3 | 0.3 |

**Supplementary Figure 3.** Comparison of Strata-X, X-Bridge and PLRP-S in terms of peak resolution (Rs) of functional and positional isomer couples**.** The plot compares Strata-X, X-Bridge, and PLRP-S cartridges across five couple of functional and positional isomers. Data points represent the Rs as detailed in the accompanying table.

Comparison of Strata-X, X-Bridge and PLRP-S in terms of peak resolution (Rs) of functional and positional isomer couples.

Rs

|  | Strata-X | X-Bridge | PLRP-S |
| --- | --- | --- | --- |
| MnBP and MiBP | 1.03529 | 0.859729 | 1.06952 |
| 5-cx-MEPTP and OH-MiNP | 1.17647 | 1.23529 | 1.12299 |
| 5-OH-MEHTP and 5-OH-MEHP | 5.13369 | 5.41176 | 5.52941 |
| OH-MPHP and cx-MiNP | 4.50304 | 5.06787 | 4.92375 |
| MnOP and MEHP | 1.6122 | 1.96078 | 1.92941 |

$$Rs=\frac{(Retention time 2-Retention time 1)}{0.85\cdot({Width1}_{50\%Height}+{Width2}_{50\%Height})}$$

**Supplementary Figure 4.** Linearity of 4-MU formation across varying 4-MUG concentrations. The regression line (y = 0.0061x + 0.0033) includes 36 measurements (n = 6 for each concentration, representing different hydrolysis times, from 0 to 240 min). The high linearity (R^2^ = 0.9996) and the overlapping data points for each concentration indicate that MUG deconjugation is instantaneous under the tested enzymatic conditions, regardless of incubation time or substrate concentration.

Linearity of 4-MU formation across varying 4-MUG concentrations

| µg/L 4-MUG | Area ratio 4-MU |
| --- | --- |
| 6.25 | 0.043 |
| 6.25 | 0.046 |
| 6.25 | 0.045 |
| 6.25 | 0.046 |
| 6.25 | 0.045 |
| 6.25 | 0.049 |
| 6.25 | 0.042 |
| 12.5 | 0.085 |
| 12.5 | 0.077 |
| 12.5 | 0.078 |
| 12.5 | 0.084 |
| 12.5 | 0.085 |
| 12.5 | 0.088 |
| 12.5 | 0.08 |
| 25 | 0.15 |
| 25 | 0.147 |
| 25 | 0.151 |
| 25 | 0.163 |
| 25 | 0.15 |
| 25 | 0.161 |
| 25 | 0.158 |
| 50 | 0.288 |
| 50 | 0.317 |
| 50 | 0.312 |
| 50 | 0.318 |
| 50 | 0.289 |
| 50 | 0.305 |
| 50 | 0.302 |
| 100 | 0.615 |
| 100 | 0.584 |
| 100 | 0.631 |
| 100 | 0.649 |
| 100 | 0.634 |
| 100 | 0.593 |
| 500 | 3.028 |
| 500 | 3.082 |
| 500 | 3.006 |
| 500 | 3.062 |
| 500 | 3.138 |
| 500 | 3.055 |
| 500 | 3.108 |

**Supplementary Figure 5.** Hydrolysis kinetics of 5-OH-MEHP in human urine. The production of the deconjugated metabolite reaches a steady state at 30 minutes, with consistent area ratios maintained up to 240 minutes.

Hydrolysis kinetics of 5-OH-MEHP in human urine

Area Ratio ± SD

| Time (Min) | Ymedia |
| --- | --- |
| 0 | 0.0304286 |
| 30 | 0.0392857 |
| 60 | 0.0372857 |
| 90 | 0.0375714 |
| 120 | 0.037 |
| 180 | 0.0363333 |
| 240 | 0.037 |

**Supplementary Table 2.** Concentrations of plasticizer metabolites in non-hydrolized pooled urine.

| Analyte | Non-hydrolized pooled urine  (µg/L) |
| --- | --- |
| MMP | 1.30 |
| MEP | 3.97 |
| 5-OH-MEHP | < 0.39 |
| 5-cx-MEPP | 0.20 |
| MiBP | < 0.78 |
| MnBP | < 0.78 |
| 5-oxo-MEHP | < 0.10 |
| OH-MiNP | < 0.20 |
| 5-OH-MEHTP | < 0.10 |
| cx-MiNP | 0.19 |
| 5-cx-MEPTP | 2.84 |
| 7-OH-MINCH | < 0.39 |
| MBzP | < 0.20 |
| MCHP | < 0.20 |
| cx-MiDP | < 0.39 |
| MnPP | < 0.10 |
| OH-MPHP | < 0.10 |
| 7-oxo-MINCH | < 0.16 |
| MnHxP | < 0.10 |
| MEHP | < 0.39 |
| MnOP | < 0.10 |

**Supplementary Table 3.** Statistical parameters of calibration curves obtained across three independent analytical sequences (batches 1-3). For each batch, the standard error of the intercept (SEq), the slope (m), the coefficient of determination (R^2^) and the calculated LLOQ are reported. The final column provides the average calculated LLOQ values. These calculated LLOQ were experimentally verified to define the LLOQ, whose values are reported in Table 4 of the main text.

|  | ***Batch 1*** | | | | ***Batch 2*** | | | | ***Batch 3*** | | | | **Average calc LLOQ**  **µg/L** |
| --- | --- | --- | --- | --- | --- | --- | --- | --- | --- | --- | --- | --- | --- |
| **Anaytes** | **SEq**  **µg/L** | **m** | **R^2^** | **Calc**  **LLOQ 1**  **µg/L** | **SEq**  **µg/L** | **m** | **R^2^** | **Calc LLOQ 2**  **µg/L** | **SEq**  **µg/L** | **m** | **R^2^** | **Calc LLOQ 3**  **µg/L** |  |
| **MMP** | 0.00087 | 0.030 | 0.9997 | 0.29 | 0.00106 | 0.037 | 0.9993 | 0.29 | 0.00295 | 0.029 | 0.9950 | 1.00 | 0.53 |
| **MEP** | 0.00105 | 0.050 | 0.9991 | 0.21 | 0.00152 | 0.063 | 0.9988 | 0.24 | 0.00264 | 0.049 | 0.9992 | 0.54 | 0.33 |
| **5-OH-MEHP** | 0.00767 | 0.264 | 0.9988 | 0.29 | 0.01064 | 0.380 | 0.9987 | 0.28 | 0.01731 | 0.251 | 0.9954 | 0.69 | 0.42 |
| **5-cx-MEPP** | 0.02227 | 0.825 | 0.9959 | 0.27 | 0.01608 | 1.237 | 0.9976 | 0.13 | 0.02846 | 0.837 | 0.9959 | 0.34 | 0.25 |
| **MiBP** | 0.00676 | 0.141 | 0.9989 | 0.48 | 0.00618 | 0.187 | 0.9990 | 0.33 | 0.00336 | 0.140 | 0.9988 | 0.24 | 0.35 |
| **MnBP** | 0.00590 | 0.107 | 0.9989 | 0.55 | 0.00548 | 0.130 | 0.9991 | 0.42 | 0.00416 | 0.107 | 0.9985 | 0.39 | 0.45 |
| **5-oxo-MEHP** | 0.00369 | 0.410 | 0.9994 | 0.09 | 0.00807 | 0.576 | 0.9983 | 0.14 | 0.00453 | 0.412 | 0.9990 | 0.11 | 0.11 |
| **OH-MiNP** | 0.20332 | 1.287 | 0.9791 | 1.58 | 0.06413 | 1.781 | 0.9974 | 0.36 | 0.06057 | 1.289 | 0.9920 | 0.47 | 0.80 |
| **5-OH-MEHTP** | 0.00896 | 1.120 | 0.9977 | 0.08 | 0.01589 | 1.589 | 0.9983 | 0.10 | 0.00791 | 1.130 | 0.9984 | 0.07 | 0.08 |
| **cx-MiNP** | 0.01705 | 0.437 | 0.9945 | 0.39 | 0.00597 | 0.597 | 0.9992 | 0.10 | 0.00762 | 0.423 | 0.9989 | 0.18 | 0.22 |
| **5-cx-MEPTP** | 0.03261 | 0.544 | 0.9969 | 0.60 | 0.05405 | 0.795 | 0.9977 | 0.68 | 0.01392 | 0.557 | 0.9977 | 0.25 | 0.51 |
| **7-OH-MiNCH** | 0.00661 | 0.348 | 0.9984 | 0.19 | 0.01994 | 0.443 | 0.9980 | 0.45 | 0.00783 | 0.340 | 0.9978 | 0.23 | 0.29 |
| **MBzP** | 0.00277 | 0.120 | 0.9989 | 0.23 | 0.00246 | 0.153 | 0.9977 | 0.16 | 0.00368 | 0.115 | 0.9979 | 0.32 | 0.24 |
| **MCHP** | 0.00175 | 0.146 | 0.9986 | 0.12 | 0.00544 | 0.202 | 0.9985 | 0.27 | 0.00177 | 0.147 | 0.9986 | 0.12 | 0.17 |
| **cx-MiDP** | 0.00360 | 0.100 | 0.9944 | 0.36 | 0.00407 | 0.140 | 0.9992 | 0.29 | 0.00204 | 0.102 | 0.9982 | 0.20 | 0.28 |
| **MnPP** | 0.00111 | 0.158 | 0.9998 | 0.07 | 0.00230 | 0.230 | 0.9996 | 0.10 | 0.00132 | 0.166 | 0.9988 | 0.08 | 0.08 |
| **OH-MPHP** | 0.00875 | 0.729 | 0.9987 | 0.12 | 0.01619 | 0.952 | 0.9987 | 0.17 | 0.00624 | 0.693 | 0.9985 | 0.09 | 0.13 |
| **7-oxo-MINCH** | 0.01160 | 0.611 | 0.9982 | 0.19 | 0.00698 | 0.581 | 0.9997 | 0.12 | 0.01161 | 0.683 | 0.9990 | 0.17 | 0.16 |
| **MnHxP** | 0.00076 | 0.252 | 0.9998 | 0.03 | 0.00245 | 0.306 | 0.9997 | 0.08 | 0.00124 | 0.247 | 0.9995 | 0.05 | 0.05 |
| **MEHP** | 0.00293 | 0.183 | 0.9988 | 0.16 | 0.00191 | 0.238 | 0.9994 | 0.08 | 0.00342 | 0.180 | 0.9984 | 0.19 | 0.14 |
| **MnOP** | 0.00207 | 0.188 | 0.9990 | 0.11 | 0.00133 | 0.222 | 0.9997 | 0.06 | 0.00275 | 0.183 | 0.9980 | 0.15 | 0.11 |

**Supplementary Table 4.** Percent extraction recovery of the on-line SPE method at three different spiked levels.

| Analyte | Level | Concentration  (µg/L) | % Recovery  (Min - Max)  n = 3 |
| --- | --- | --- | --- |
| MMP | Spike 1 | 3.13 | 94 (73 - 111) |
|  | Spike 2 | 12.50 | 106 (98 - 113) |
|  | Spike 3 | 50.00 | 101 (89 - 107) |
| MEP | Spike 1 | 6.25 | 104 (98 - 109) |
|  | Spike 2 | 12.50 | 105 (98 - 112) |
|  | Spike 3 | 50.00 | 100 (96 - 103) |
| 5-OH-MEHP | Spike 1 | 1.56 | 97 (91 - 105) |
|  | Spike 2 | 12.50 | 97 (97 - 98) |
|  | Spike 3 | 50.00 | 101 (94 - 104) |
| 5-cx-MEPP | Spike 1 | 0.78 | 103 (92 - 115) |
|  | Spike 2 | 6.25 | 94 (88 - 100) |
|  | Spike 3 | 50.00 | 100 (91 - 118) |
| MiBP | Spike 1 | 3.13 | 100 (94 - 110) |
|  | Spike 2 | 12.50 | 107 (98 - 114) |
|  | Spike 3 | 50.00 | 101 (96 - 106) |
| MnBP | Spike 1 | 3.13 | 104 (100 - 107) |
|  | Spike 2 | 12.50 | 102 (94 - 108) |
|  | Spike 3 | 50.00 | 104 (100 - 107) |
| 5-oxo-MEHP | Spike 1 | 0.39 | 112 (108 - 118) |
|  | Spike 2 | 3.13 | 108 (103 - 118) |
|  | Spike 3 | 50.00 | 101 (93 - 108) |
| OH-MiNP | Spike 1 | 0.78 | 109 (100 - 114) |
|  | Spike 2 | 6.25 | 98 (87 - 105) |
|  | Spike 3 | 50.00 | 104 (102 - 105) |
| 5-OH-MEHTP | Spike 1 | 0.39 | 102 (86 - 122) |
|  | Spike 2 | 3.13 | 99 (93 - 110) |
|  | Spike 3 | 50.00 | 95 (88 - 109) |
| cx-MiNP | Spike 1 | 1.56 | 103 (99 - 108) |
|  | Spike 2 | 12.50 | 102 (101 - 103) |
|  | Spike 3 | 50.00 | 95 (88 - 100) |
| 5-cx-MEPTP | Spike 1 | 3.13 | 90 (83 - 96) |
|  | Spike 2 | 12.50 | 101 (94 - 113) |
|  | Spike 3 | 50.00 | 103 (99 - 107) |
| 7-OH-MINCH | Spike 1 | 1.56 | 103 (98 - 109) |
|  | Spike 2 | 12.50 | 102 (92 - 106) |
|  | Spike 3 | 50.00 | 106 (93 - 114) |
| MBzP | Spike 1 | 0.78 | 95 (89 - 101) |
|  | Spike 2 | 6.25 | 98 (95 - 103) |
|  | Spike 3 | 50.00 | 108 (100 - 116) |
| MCHP | Spike 1 | 0.78 | 95 (88 - 99) |
|  | Spike 2 | 6.25 | 100 (99 - 100) |
|  | Spike 3 | 50.00 | 106 (104 - 110) |
| cx-MiDP | Spike 1 | 1.56 | 104 (92 - 113) |
|  | Spike 2 | 12.50 | 96 (86 - 102) |
|  | Spike 3 | 50.00 | 93 (87 - 101) |
| MnPP | Spike 1 | 0.78 | 103 (94 - 117) |
|  | Spike 2 | 6.25 | 96 (88 - 101) |
|  | Spike 3 | 50.00 | 97 (96 - 99) |
| OH-MPHP | Spike 1 | 0.39 | 102 (95 - 111) |
|  | Spike 2 | 6.25 | 99 (97 - 102) |
|  | Spike 3 | 50.00 | 94 (87 - 100) |
| MnHxP | Spike 1 | 0.78 | 105 (99 - 111) |
|  | Spike 2 | 6.25 | 94 (90 - 98) |
|  | Spike 3 | 50.00 | 106 (95 - 117) |
| MEHP | Spike 1 | 1.56 | 98 (91 - 104) |
|  | Spike 2 | 12.50 | 103 (97 - 111) |
|  | Spike 3 | 50.00 | 94 (89 - 96) |
| MnOP | Spike 1 | 0.39 | 94 (76 - 107) |
|  | Spike 2 | 6.25 | 102 (101 - 103) |
|  | Spike 3 | 50.00 | 98 (94 - 100) |

**Supplementary Table 5.** Concentrations of plasticizer metabolites in the six individual non-hydrolyzed urines used as blank matrices to perform matrix effect test.

| Analyte | Average Conc. µg/L (Min - Max)  n = 6 |
| --- | --- |
| MMP | 2.38 (1.34 - 4.64) |
| MEP | 32.57 (3.38 - 87.03) |
| 5-OH-MEHP | 0.91 (0.80 - 0.99) |
| 5-cx-MEPP | 0.61 (0.29 - 1.29) |
| MiBP | < 0.78 (< 0.78 - 0.89) |
| MnBP | 1.58 (1.19 - 2.01) |
| 5-oxo-MEHP | < 0.10 |
| OH-MiNP | < 0.20 |
| 5-OH-MEHTP | 0.11 (< 0.10 - 0.41) |
| cx-MiNP | 0.68 (0.16 - 1.85) |
| 5-cx-MEPTP | 10.50 (0.80 - 41.66) |
| 7-OH-MINCH | < 0.39 |
| MBzP | < 0.20 |
| MCHP | < 0.20 |
| cx-MiDP | < 0.39 |
| MnPP | < 0.10 |
| OH-MPHP | < 0.10 |
| 7-oxo-MINCH | < 0.16 |
| MnHxP | < 0.10 |
| MEHP | < 0.39 |
| MnOP | < 0.10 |

**Supplementary Figure 6.** Degree of glucuronidation of plasticizer metabolites in human urine. The blue bars represent the mean percentage of conjugated form relative to the total metabolite concentration, calculated by comparing non-hydrolyzed and hydrolyzed urine samples collected from six volunteers (n = 6). The error bars represent the standard deviation (± SD), expressing the inter-individual variability.

degree of glucuronidation of plasticizer metabolites

% conjugated metabolite ± sd

|  | QCL_stabilità |
| --- | --- |
| MEP | 28.2 |
| 5-OH-MEHP | 96.9 |
| 5-cx-MEPP | 48.3 |
| MiBP | 94.5 |
| MnBP | 95.9 |
| 5-oxo MEHP | 96.1 |
| OH-MiNP | 91.1 |
| 5-OH-MEHTP | 59.3 |
| cx-MiNP | 39 |
| 5-cx-MEPTP | 7 |
| 7-OH-MINCH | 100 |
| MBzP | 97.5 |
| MEHP | 88.3 |
| 7-oxo-MINCH | 98.1 |

**Supplementary Figure 7.** Intra-run precision at QC and LLOQ levels in a graphical representation.

Intra-run precision and lloq precision

% RELATIVE STANDARD DEVIATION

|  | QCL | QCM | QCH | Max. relative standard deviation: 15% | % Relative standard deviation of double measurements | LLOQ precision | Max. relative standard deviation at LLOQ: 20% |
| --- | --- | --- | --- | --- | --- | --- | --- |
| MMP | 3.6 | 3.1 | 1.8 | 15 |  | 12.6 | 20 |
| MEP | 4 | 2.9 | 2.1 | 15 |  | 6 | 20 |
| 5-OH-MEHP | 2.5 | 3 | 1.7 | 15 |  | 7.9 | 20 |
| 5-cx-MEPP | 4.5 | 4.8 | 3.6 | 15 |  | 6.3 | 20 |
| MiBP | 4.9 | 3.9 | 3.8 | 15 |  | 12.5 | 20 |
| MnBP | 13.8 | 6.6 | 2.1 | 15 |  | 17.9 | 20 |
| 5-oxo MEHP | 2.9 | 1.8 | 2.4 | 15 |  | 4.5 | 20 |
| OH-MiNP | 3.9 | 3.8 | 3.3 | 15 | 7 | 6 | 20 |
| 5-OH-MEHTP | 4.6 | 3.1 | 3.3 | 15 |  | 4.7 | 20 |
| cx-MiNP | 8.5 | 2.9 | 2 | 15 | 3.4 | 9 | 20 |
| 5-cx-MEPTP | 5.8 | 5.3 | 2.3 | 15 |  | 10.2 | 20 |
| 7-OH-MINCH | 3.6 | 2.7 | 2.7 | 15 | 5.6 | 3.8 | 20 |
| MBzP | 7.4 | 4.3 | 3.5 | 15 |  | 6.5 | 20 |
| MCHP | 10.8 | 4.3 | 3.6 | 15 |  | 7.8 | 20 |
| cx-MiDP | 8.5 | 3.4 | 5.3 | 15 | 3.7 | 2.4 | 20 |
| MnPP | 5 | 2.2 | 2.8 | 15 |  | 6.6 | 20 |
| OH-MPHP | 1.8 | 3.1 | 4.8 | 15 | 6.8 | 2.8 | 20 |
| 7-oxo-MINCH | 1 | 1.4 | 3.3 | 15 | 13.7 |  | 20 |
| MnHxP | 4.1 | 2.8 | 2.5 | 15 |  | 2.5 | 20 |
| MEHP | 2.9 | 2.4 | 3 | 15 |  | 11.8 | 20 |
| MnOP | 3.7 | 3.5 | 2.3 | 15 |  | 4 | 20 |

**Supplementary Figure 8.** Between-run precision at QC levels in a graphical representation.

bETWEEN-RUN PRECISION

% relative standard deviation

|  | QCL | QCM | QCH | Max. relative standard deviation: 15% |
| --- | --- | --- | --- | --- |
| MMP | 6.1 | 4 | 2.7 | 15 |
| MEP | 4.2 | 4.8 | 2.4 | 15 |
| 5-OH-MEHP | 3.3 | 3.8 | 2 | 15 |
| 5-cx-MEPP | 5.4 | 5.6 | 5.3 | 15 |
| MiBP | 5.3 | 3.9 | 3.9 | 15 |
| MnBP | 15.6 | 7.1 | 2 | 15 |
| 5-oxo MEHP | 5.7 | 2.8 | 2.9 | 15 |
| OH-MiNP | 4 | 6.3 | 5.9 | 15 |
| 5-OH-MEHTP | 6 | 3.4 | 3.8 | 15 |
| cx-MiNP | 9.3 | 4.9 | 2.5 | 15 |
| 5-cx-MEPTP | 12.3 | 6 | 5.4 | 15 |
| 7-OH-MINCH | 4.7 | 3.1 | 3.7 | 15 |
| MBzP | 9.1 | 4.3 | 4.8 | 15 |
| MCHP | 13.4 | 5.4 | 3.9 | 15 |
| cx-MiDP | 8.4 | 6.7 | 6.2 | 15 |
| MnPP | 5.9 | 4.5 | 5.5 | 15 |
| OH-MPHP | 4.3 | 3.6 | 5.9 | 15 |
| MnHxP | 5.7 | 2.8 | 2.5 | 15 |
| MEHP | 6.7 | 3.3 | 2.9 | 15 |
| MnOP | 5.8 | 5.7 | 2.4 | 15 |

**Supplementary Figure 9.** Between-run accuracy at QC levels and intra-run accuracy at LLOQ levels in a graphical representation.

between-run ACCURACY and LLOQ ACCURACY

%ACCURACY

|  | QCL | QCM | QCH | Inferior and superior limits of accuracy: 85%-115% | Superior limit of accuracy 115% | Target | LLOQ Accuracy | Inferior and superior limits of accuracy at LLOQ: 80%-120% | Superior limit of accuracy: 120% |
| --- | --- | --- | --- | --- | --- | --- | --- | --- | --- |
| MMP | 105.4 | 104.1 | 100.6 | 85 | 115 | 100 | 89.2 | 80 | 120 |
| MEP | 105.1 | 104.4 | 100.3 | 85 | 115 | 100 | 115.2 | 80 | 120 |
| 5-OH-MEHP | 105 | 99.9 | 100.1 | 85 | 115 | 100 | 111.4 | 80 | 120 |
| 5-cx-MEPP | 98.3 | 102.3 | 102.6 | 85 | 115 | 100 | 101.1 | 80 | 120 |
| MiBP | 103.6 | 105.1 | 102.7 | 85 | 115 | 100 | 101.2 | 80 | 120 |
| MnBP | 102.9 | 103.2 | 101.2 | 85 | 115 | 100 | 117 | 80 | 120 |
| 5-oxo MEHP | 104.2 | 106 | 101.5 | 85 | 115 | 100 | 103.4 | 80 | 120 |
| OH-MiNP | 96.5 | 104.3 | 105.2 | 85 | 115 | 100 | 93.8 | 80 | 120 |
| 5-OH-MEHTP | 100.8 | 104.9 | 100.1 | 85 | 115 | 100 | 101.8 | 80 | 120 |
| cx-MiNP | 104.8 | 107.1 | 100.5 | 85 | 115 | 100 | 107.2 | 80 | 120 |
| 5-cx-MEPTP | 105.9 | 107.5 | 100.8 | 85 | 115 | 100 | 101.6 | 80 | 120 |
| 7-OH-MINCH | 105.1 | 97.2 | 103 | 85 | 115 | 100 | 112.8 | 80 | 120 |
| MBzP | 99.3 | 101 | 100.8 | 85 | 115 | 100 | 109.7 | 80 | 120 |
| MCHP | 102 | 100.8 | 99 | 85 | 115 | 100 | 117.3 | 80 | 120 |
| cx-MiDP | 99.3 | 104.1 | 96.5 | 85 | 115 | 100 | 112.9 | 80 | 120 |
| MnPP | 98.3 | 99.8 | 98.9 | 85 | 115 | 100 | 110.3 | 80 | 120 |
| OH-MPHP | 105.5 | 105.1 | 100.8 | 85 | 115 | 100 | 113.6 | 80 | 120 |
| 7-oxo-MINCH* | 105.1 | 108.5 | 107.8 | 85 | 115 | 100 |  | 80 | 120 |
| MnHxP | 103.8 | 103 | 102.3 | 85 | 115 | 100 | 118.7 | 80 | 120 |
| MEHP | 105.6 | 103.9 | 101.1 | 85 | 115 | 100 | 95.8 | 80 | 120 |
| MnOP | 100 | 103.6 | 101.5 | 85 | 115 | 100 | 111.5 | 80 | 120 |

*Data from intra-run test.

**Supplementary Figure 10.** Long-term stability at QCL level in a graphical representation.

six-Month stability

% Accuracy

|  | QCL | Inferior and superior limits of stability: 85%-115% | Superior limit of stability 115% | Target |
| --- | --- | --- | --- | --- |
| MMP | 93 | 85 | 115 | 100 |
| MEP | 94 | 85 | 115 | 100 |
| 5-OH-MEHP | 96 | 85 | 115 | 100 |
| 5-cx-MEPP | 93 | 85 | 115 | 100 |
| MiBP | 89 | 85 | 115 | 100 |
| MnBP | 94 | 85 | 115 | 100 |
| 5-oxo MEHP | 100 | 85 | 115 | 100 |
| OH-MiNP | 90 | 85 | 115 | 100 |
| 5-OH-MEHTP | 93 | 85 | 115 | 100 |
| cx-MiNP | 99 | 85 | 115 | 100 |
| 5-cx-MEPTP | 105 | 85 | 115 | 100 |
| 7-OH-MINCH | 91 | 85 | 115 | 100 |
| MBzP | 94 | 85 | 115 | 100 |
| MCHP | 86 | 85 | 115 | 100 |
| cx-MiDP | 98 | 85 | 115 | 100 |
| MnPP | 89 | 85 | 115 | 100 |
| OH-MPHP | 91 | 85 | 115 | 100 |
| MnHxP | 89 | 85 | 115 | 100 |
| MEHP | 96 | 85 | 115 | 100 |
| MnOP | 85 | 85 | 115 | 100 |

**Supplementary Figure 11.** Percent extraction recovery of the on-line SPE method at three different spiked levels in a graphical representation.

EXTRACTION RECOVERY

ExtRACTION RECOVERY %

|  | Spike 1 | Spike 2 | Spike 3 | Target |
| --- | --- | --- | --- | --- |
| MMP | 94 | 106 | 101 | 100 |
| MEP | 104 | 105 | 100 | 100 |
| 5-OH-MEHP | 97 | 97 | 101 | 100 |
| 5-cx-MEPP | 103 | 94 | 100 | 100 |
| MiBP | 100 | 107 | 101 | 100 |
| MnBP | 104 | 102 | 104 | 100 |
| 5-oxo MEHP | 112 | 108 | 101 | 100 |
| OH-MiNP | 109 | 98 | 104 | 100 |
| 5-OH-MEHTP | 102 | 99 | 95 | 100 |
| cx-MiNP | 103 | 102 | 95 | 100 |
| 5-cx-MEPTP | 90 | 101 | 103 | 100 |
| 7-OH-MINCH | 103 | 102 | 106 | 100 |
| MBzP | 95 | 98 | 108 | 100 |
| MCHP | 95 | 100 | 106 | 100 |
| cx-MiDP | 104 | 96 | 93 | 100 |
| MnPP | 103 | 96 | 97 | 100 |
| OH-MPHP | 102 | 99 | 94 | 100 |
| MnHxP | 105 | 94 | 106 | 100 |
| MEHP | 98 | 103 | 94 | 100 |
| MnOP | 94 | 102 | 98 | 100 |

**Supplementary Figure 12.** Inter-individual matrix effect for plasticizer metabolites in human urine. Data represent the dispersion (expressed as % RSD) of calibration slopes and three spiked levels obtained from six individual calibration curves.

Matrix effect expressed as inter-individual dispersion of spiked samples and calibration slopes

% relative standard deviation

|  | Level 1 | Level 2 | Level 3 | RSD_slopes limit for absence of inter-individual matrix effect: 5% | RSD limit for inter-individual dispersion of QC: 15% | % RSD_slopes | RSD_slopes, limit for inter-individual matrix effect of minor impact: 10%" |
| --- | --- | --- | --- | --- | --- | --- | --- |
| MMP | 18.8 | 11.6 | 4.6 | 5 | 15 | 4.4 | 10 |
| MEP | 19.1 | 9.3 | 4.9 | 5 | 15 | 4.8 | 10 |
| 5-OH-MEHP | 13.9 | 13.6 | 9.8 | 5 | 15 | 9.3 | 10 |
| 5-cx-MEPP | 4.9 | 5.3 | 4.8 | 5 | 15 | 5.1 | 10 |
| MiBP | 3.8 | 6.7 | 2.6 | 5 | 15 | 3.5 | 10 |
| MnBP | 25.3 | 5.3 | 6.6 | 5 | 15 | 6.8 | 10 |
| 5-oxo MEHP | 5.1 | 4.3 | 7.1 | 5 | 15 | 8 | 10 |
| OH-MiNP | 15.1 | 10.7 | 5.4 | 5 | 15 | 5.1 | 10 |
| 5-OH-MEHTP | 17.7 | 3.9 | 6.8 | 5 | 15 | 5 | 10 |
| cx-MiNP | 15.4 | 4.8 | 6.9 | 5 | 15 | 4.1 | 10 |
| 5-cx-MEPTP | 17.9 | 6.2 | 7.3 | 5 | 15 | 6.1 | 10 |
| 7-OH-MINCH | 6.6 | 5.1 | 4.6 | 5 | 15 | 4.6 | 10 |
| MBzP | 22.2 | 13.1 | 15.4 | 5 | 15 | 13.9 | 10 |
| MCHP | 11.5 | 12.1 | 8.1 | 5 | 15 | 7.8 | 10 |
| cx-MiDP | 16.7 | 16.3 | 14.2 | 5 | 15 | 13.6 | 10 |
| MnPP | 5 | 7.7 | 3.6 | 5 | 15 | 3.5 | 10 |
| OH-MPHP | 6.5 | 2 | 7.2 | 5 | 15 | 2.5 | 10 |
| 7-oxo-MINCH |  |  |  | 5 | 15 | 2 | 10 |
| MnHxP | 7.1 | 9.3 | 3.7 | 5 | 15 | 2.7 | 10 |
| MEHP | 14.7 | 2 | 2.4 | 5 | 15 | 1.8 | 10 |
| MnOP | 10.2 | 5.2 | 3 | 5 | 15 | 1.9 | 10 |

**Supplementary Figure 13.** Matrix effect expressed as inter-subject mean accuracy of spiked samples. Each data point represents the mean accuracy (%) calculated across urine samples from six different volunteers (n = 6) at three fortification levels (Level 1, 2, and 3).

Matrix effect as inter-subject mean accuracy of spiked samples

% ACCURACY

|  | Level 1 | Level 2 | Level 3 | Inferior and superior limit of inter-individual mean accuracy: 85%-115% | Superior limit of inter-individual mean accuracy: 115% | Target |
| --- | --- | --- | --- | --- | --- | --- |
| MMP | 108 | 97 | 101 | 85 | 115 | 100 |
| MEP | 93 | 97 | 100 | 85 | 115 | 100 |
| 5-OH-MEHP | 107 | 98 | 101 | 85 | 115 | 100 |
| 5-cx-MEPP | 104 | 102 | 101 | 85 | 115 | 100 |
| MiBP | 99 | 98 | 101 | 85 | 115 | 100 |
| MnBP | 84 | 94 | 99 | 85 | 115 | 100 |
| 5-oxo MEHP | 92 | 99 | 101 | 85 | 115 | 100 |
| OH-MiNP | 90 | 100 | 103 | 85 | 115 | 100 |
| 5-OH-MEHTP | 97 | 102 | 98 | 85 | 115 | 100 |
| cx-MiNP | 102 | 103 | 103 | 85 | 115 | 100 |
| 5-cx-MEPTP | 111 | 103 | 99 | 85 | 115 | 100 |
| 7-OH-MINCH | 89 | 96 | 102 | 85 | 115 | 100 |
| MBzP | 102 | 95 | 101 | 85 | 115 | 100 |
| MCHP | 98 | 97 | 102 | 85 | 115 | 100 |
| cx-MiDP | 104 | 95 | 100 | 85 | 115 | 100 |
| MnPP | 94 | 104 | 100 | 85 | 115 | 100 |
| OH-MPHP | 104 | 102 | 97 | 85 | 115 | 100 |
| MnHxP | 102 | 99 | 101 | 85 | 115 | 100 |
| MEHP | 96 | 99 | 99 | 85 | 115 | 100 |
| MnOP | 86 | 92 | 100 | 85 | 115 | 100 |

**Supplementary Figure 14.** Performance in the G-EQUAS proficiency testing scheme (Rounds 74 and 75). The plots show the measured concentrations (diamonds) of the urinary biomarkers relative to the consensus target values (green) and the tolerance intervals (red) for both low (A) and high (B) concentration levels. The x-axis indicates the G-EQUAS proficiency testing rounds (74 and 75), while the y-axis represents the concentrations expressed in µg/L. Blue diamonds represent results within the tolerance range, whereas the orange diamonds (OH-MiNP, Round 74, levels A and B) indicate a value outside the assigned interval.
